# Breaking Trade‐Off between Selectivity and Activity of Nickel‐Based Hydrogenation Catalysts by Tuning Both Steric Effect and d‐Band Center

**DOI:** 10.1002/advs.201900054

**Published:** 2019-03-25

**Authors:** Ruijie Gao, Lun Pan, Huiwen Wang, Yunduo Yao, Xiangwen Zhang, Li Wang, Ji‐Jun Zou

**Affiliations:** ^1^ Key Laboratory for Green Chemical Technology of the Ministry of Education School of Chemical Engineering and Technology Tianjin University Tianjin 300072 China; ^2^ Collaborative Innovative Center of Chemical Science and Engineering (Tianjin) Tianjin 300072 China

**Keywords:** d‐band center, Ni_2_P, selective hydrogenation, steric effects, thiol‐arrays

## Abstract

For selective hydrogenation of chemicals the high selectivity is always at the expense of activity and improving both selectivity and activity is challenging. Here, by chelating with *p*‐fluorothiophenol (SPhF)‐arrays, both steric and electronic effects are created to boost the performance of cheap nickel‐based catalysts. Compared with dinickel phosphide, the SPhF‐chelated one exhibits nearly 12 times higher activity and especially its selectivity is increased from 38.1% and 21.3% to nearly 100% in hydrogenations of 3‐nitrostyrene and cinnamaldehyde. Commercial catalysts like Raney Ni chelating with SPhF‐array also exhibits an enhanced selectivity from 20.5% and 23.4% to ≈100% along with doubled activity. Both experimental and density functional theory (DFT) calculation prove that the superior performance is attributed to the confined flat adsorption by ordered SPhF‐arrays and downshifted d‐band center of catalysts, leading to prohibited hydrogenation of the vinyl group and accelerative H_2_ activation. Such a surface modification can provide an easily‐realized and low‐cost way to design catalysts for the selective hydrogenation.

## 1 Introduction

Selective hydrogenation of terminal groups of chemicals containing conjugated alkene bond (e.g., nitrostyrene^1^, ^2^, ^3^, ^4^, ^5^, ^6^ and cinnamaldehyde^7^, ^8^, ^9^, ^10^) is a vital process in industrial manufacture of fine chemicals, pharmaceuticals, nutraceuticals, and agrochemicals. Nevertheless, it is difficult to avoid the hydrogenation of vinyl group due to the thermodynamic favor over the hydrogenation of aldehyde or nitro group.^11^, ^12^ Actually, conjugated vinyl group can only be adsorbed via a flat configuration for subsequent hydrogenation.^13^, ^14^, ^15^ Surface‐modification by organic molecules as a means to improve the selectivity has been successfully exploited.^7^, ^16^, ^17^, ^18^, ^19^, ^20^, ^21^ In these cases, organic molecules possessing a long branch chain and a metallophilic group are usually employed, where the metallophilic groups bind strongly with catalysts and the branch chains spread outside to form well‐defined arrays. The interchain distance is adjustable and can limit the flat adsorption of reactant when it is smaller than the reactant molecules. For instance, PtCo/amine^13^ and Pt/thiol^16^ show enhanced selectivity of –C=O in hydrogenation of cinnamaldehyde. However, due to the suppressed diffusion and adsorption of reactants by the long aliphatic chain and shielded active sites, the high selectivity is usually at the expense of activity, so the trade‐off between the activity and selectivity always exists.^13^, ^16^, ^17^, ^18^, ^19^, ^20^, ^21^ And the rare reserve and high expense further limit the large‐scale application of noble metal catalysts. Therefore, how to improve both the selectivity and activity, and use low‐cost catalysts remain a big challenge.

Dinickel phosphide (Ni_2_P) is a promising catalyst to replace noble metal catalysts owing to its Pt‐like electronic structure and low‐cost price, and shows considerable activity in the hydrogenation of vinyl, aldehyde, and nitro groups.^22^, ^23^, ^24^, ^25^, ^26^ However, the poor selectivity of Ni_2_P toward –NO_2_ and –CHO is still an open question, due to a preferential flat adsorption of nitrostyrene and cinnamaldehyde. We speculate to utilize *p*‐Fluorothiophenol (SPhF) to tune the surface of Ni_2_P because its branch chain (–PhF) can provide a considerable steric hindrance to constrain the adsorption configuration of reactants, and expect to achieve high selectivity like the case of noble metals. The key point here is that the length of –PhF is less than that of most used aliphatic chain, thus avoiding the suppression on diffusion and adsorption of reactants. And the Van der Waals interaction between benzene rings of SPhF and reactants may further enhance the adsorption of reactants. More importantly, the electronegative elements of organic molecules are expected to push the d‐band center of Ni away from the Fermi level,^27^, ^28^, ^29^, ^30^, ^31^, ^32^ which can lower the antibonding energy level and consequently strengthen the interaction between Ni_2_P and H_2_, beneficial for hydrogen activation.^33^, ^34^, ^35^, ^36^ Therefore, Ni‐based catalysts chelated by SPhF‐array may achieve the goal of hitting two birds with one stone, namely, boost both the selectivity and activity simultaneously, which provides the possibility to break the trade‐off between activity and selectivity for hydrogenation of chemicals containing conjugated alkene bond.

With these considerations, here we report that low‐cost Ni_2_P chelated with SPhF arrays (SPhF‐Ni_2_P) exhibits significantly improved selectivity and activity in hydrogenation reactions. Compared with Ni_2_P, such a metal–organic interface generates considerable steric and electronic benefits. On steric level, the upstanding thiol‐array can prevent the flat adsorption of conjugated C=C and thus avoid its reduction. On electronic level, the electron‐withdrawing thiols can efficiently downshift the d‐band center of Ni_2_P, which accelerates hydrogen activation to enhance the activity. Consequently, compared with Ni_2_P, SPhF‐Ni_2_P delivers nearly 12 time higher activity and selectivity increasing from 38.1% and 21.3% to 100% in the hydrogenation of 3‐nitrostyrene and cinnamaldehyde, outperforming reported state‐of‐the‐art catalysts. We also extend this strategy to commercial Raney Ni catalyst and achieve a selectivity increasing from 20.5% and 23.4% to 100% and 99.2% along with a doubled activity.

## 2 Results

### 2.1 Catalyst Synthesis Strategy

**Figure 1:**
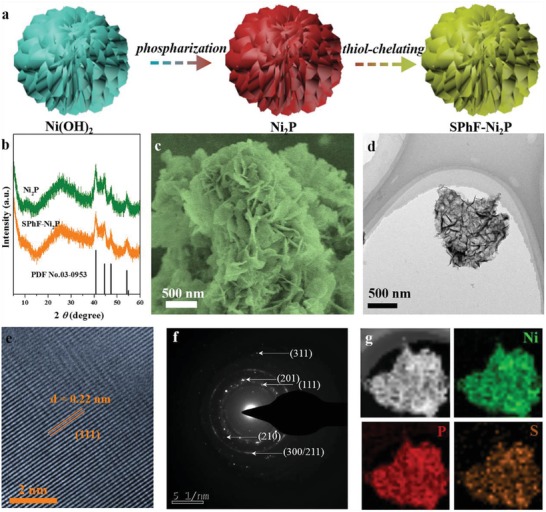
a) Schematic representation of the synthesis of SPhF‐Ni_2_P; b) XRD patterns of Ni_2_P; c) SEM, d) Low‐resolution TEM and e) HRTEM images and f) the corresponding SAED pattern of SPhF‐Ni_2_P. g) HAADF‐STEM image and element mapping images of Ni, P, and S.

Ultrathin Ni_2_P nanosheets were chosen as the substrate because a high proportion of exposed surface Ni atoms can provide exact information about SPhF‐Ni_2_P interface, helping us to clarify the intrinsic catalytic mechanism. SPhF‐Ni_2_P was synthesized by a two‐step process, as illustrated in **Figure** **1**a. First, as‐synthesized Ni(OH)_2_ (Figure S1, Supporting Information) was phosphatized by NaH_2_PO_2_ under low temperature to get hexagonal Ni_2_P phase (PDF No. 03‐0953), as revealed by X‐ray diffraction (XRD) pattern (Figure 1b, Figure S2, Supporting Information). Ni_2_P was then mixed with *p*‐Fluorothiophenol in *N, N*‐dimethylformamide at 80 °C and centrifuged to collect SPhF‐Ni_2_P. During this treatment, the strong interaction between S and Ni atoms contributes to a stable chelate. Fourier‐transform infrared spectroscopy (FT‐IR) spectra of SPhF‐Ni_2_P shows several peaks at 2992, 1396, and 690–750 cm^−1^ assigned to the C–H stretching, C–F bending, and C–H vibration of benzene ring,^37^, ^38^ confirming SPhF is chelating on the surface (Figure S3, Supporting Information). And the peaks at 11.6° and 23.7° indicate the presence of porous‐layer structure,^39^ which is further confirmed by scanning electron microscopy (SEM) and Transmission electron microscopy (TEM) images (Figure 1c–e, Figure S4, Supporting Information). The Brunauer–Emmett–Teller (BET) area of SPhF‐Ni_2_P (256.7 m^2^ g^−1^) is similar with that of Ni_2_P (251.2 m^2^ g^−1^) (Figure S5, Supporting Information). The high‐resolution TEM (HRTEM) image of SPhF‐Ni_2_P displays well‐resolved lattice fringes with an interplanar distance of 0.22 nm, assigned to the (111) plane of Ni_2_P (Figure 1e).^40^, ^41^, ^42^ The selective area electron diffraction (SEAD) pattern shows several bright rings consisted of discrete spots, indexed to different planes of hexagonal Ni_2_P (Figure 1f). Energy dispersive X‐ray (EDX) and X‐ray photoelectron spectroscopy (XPS) spectra of SPhF‐Ni_2_P indicate the Ni/P atomic ratio is around 2, close to the stoichiometry of Ni_2_P (Figures S6,S7, Supporting Information ). As shown in Figure 1g, the high‐angle angular dark‐field (HAADF) image and EDX element mapping show a homogeneous distribution of Ni, P, and S, suggesting SPhF molecules are uniformly chelating on Ni_2_P. Other catalysts with different SPhF coverages (9.5%, 19.3%, and 26.4%, detailed method in Table S1 in the Supporting Information) were also synthesized by altering the dosage of SPhF, showing similar morphology and structure with Ni_2_P and SPhF‐Ni_2_P (Figures S8,S9, Supporting Information ).

### 2.2 Simultaneously Improved Activity and Selectivity

**Figure 2:**
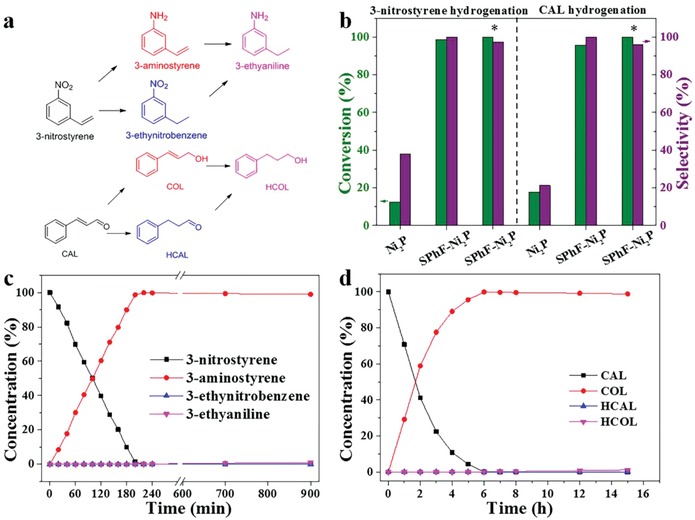
a) Possible hydrogenation route of 3‐nitrostyrene and cinnamaldehyde; b) Catalytic performances for selective hydrogenation of 3‐nitrostyrene and cinnamaldehyde over Ni_2_P and SPhF‐Ni_2_P (reaction condition: 70 °C, 200 min for 3‐nitrostyrene and 5 h for CAL; *reaction temperature: 120 °C); c) Product distribution of 3‐nitrostyrene hydrogenation catalyzed by SPhF‐Ni_2_P; d) Product distribution of CAL hydrogenation catalyzed by SPhF‐Ni_2_P.

The selective hydrogenation of 3‐nitrostyrene and cinnamaldehyde (CAL) were tested using SPhF‐Ni_2_P and Ni_2_P. As illustrated in **Figure** **2**a, the target products, i.e., 3‐aminostyrene and cinnamylalcohol (COL), are the hydrogenated intermediates, and there are possible side‐reactions yielding 3‐ethynitrobenzene and HCAL and even complete hydrogenation products. As shown in Figure 2b, Figures S10 and S11 in the Supporting Information, SPhF‐Ni_2_P exhibits a perfect selectivity of ≈100% toward 3‐aminostyrene and COL at conversion of ≈98.7% and ≈95.7% at 70 °C. When the reaction temperature is raised to 120 °C, the selectivity of 97.1% (3‐aminostyrene) and 95.8% (COL) is still perfectly preserved (Figure 2b). Figure 2c shows the target product presents a linear increase with reaction time, accompanied by a linearly decreasing concentration of 3‐nitrostyrene (MS data is provided in Figure S12, Supporting Information). Even when the reaction is prolonged to 900 min or mixture of nitrobenzene and styrene is tested (Figure S13, Supporting Information), the selectivity to nitro group is maintained at ≥99%. Likewise, CAL is also high selectively hydrogenated to COL (≈99%) until reaching a full conversion (Figure 2d). In comparison, under the same condition, Ni_2_P shows a very low activity (conversion of 12.5% and 38.1%). And it gives an extremely low selectivity (21.3% and 17.8%), yielding a large amount of 3‐ethylnitrobenzene and HCAL, suggesting a thermodynamic favoring for the hydrogenation of –C=C over Ni_2_P. These results obviously show the thiol chelation generates a switch from hydrogenation of –C=C to hydrogentaion of –NO_2_ and –CHO. Besides, kinetic curves show both conversions of 3‐nitrostyrene and cinnamaldehyde increase linearly depend on reaction time, suggesting two reactions on SPhF‐Ni_2_P follow pseudo‐zero‐order kinetics, and the rate determining step should be H_2_ activation.

**Figure 3:**
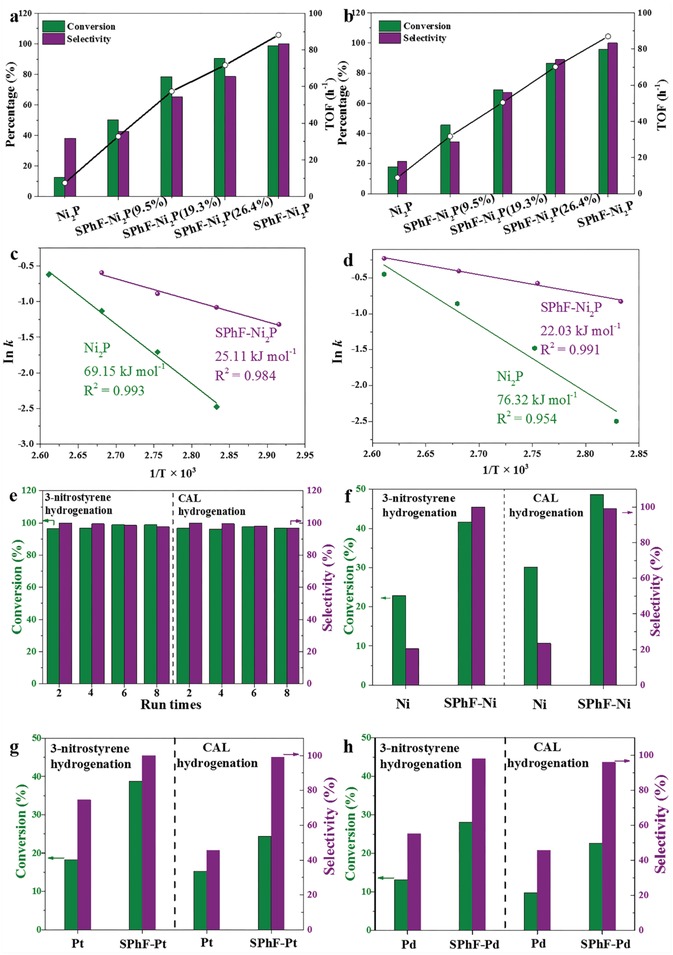
Catalytic performances for selective hydrogenation of a) 3‐nitrostyrene and b) cinnamaldehyde over Ni_2_P and SPhF‐Ni_2_P with different SPhF coverages (reaction condition: 70 °C, 200 min for 3‐nitrostyrene and 5 h for CAL); The TOF value was measured at 1 h. Arrhenius plot for the hydrogenation of c) 3‐nitrostyrene and d) cinnamaldehyde over Ni_2_P and SPhF‐Ni_2_P catalysts. e) Recycling test of SPhF‐Ni_2_P for hydrogenation of 3‐nitrostyrene and cinnamaldehyde. f) Catalytic performances for hydrogenation of 3‐nitrostyrene and cinnamaldehyde over Raney Ni and SPhF‐Ni (reaction condition: 120 °C, 200 min for 3‐nitrostyrene and 5 h for CAL). Catalytic performances for hydrogenation of 3‐nitrostyrene and cinnamaldehyde over g) Pt and SPhF‐Pt, h) Pd and SPhF‐Pd. (reaction condition: 40 °C, 10 min for 3‐nitrostyrene and 70 °C, 1 h for cinnamaldehyde in the case of Pt; 70 °C, 1 h for 3‐nitrostyrene and 100 °C, 1 h for cinnamaldehyde in the case of Pd).

The SPhF chelation also significantly enhances the hydrogenation activity. As shown in **Figure** **3**a,b, Figures S10, S11, and Tables S2, S3 in the Supporting Information, the turn over frequency (TOF) value of SPhF‐Ni_2_P is 12 time and 10 time higher than that of Ni_2_P (88.2 vs 7.4 h^−1^ and 87 vs 8.9 h^−1^ for hydrogenation of 3‐nitrostyrene and CAL), and the reaction rate of SPhF‐Ni_2_P is also 10 time higher than that of Ni_2_P (0.50 vs 0.06 mol L^−1^ min^−1^ and 0.296 vs 0.044 mol L^−1^ h^−1^ for hydrogenation of 3‐nitrostyrene and CAL). SPhF‐Ni_2_P requires a significantly lower activation energy than Ni_2_P (24.11 vs 69.15 and 22.03 vs 76.32 kJ mol^−1^) as shown in Figure 3c,d. The dependency of the hydrogenation performance on SPhF coverage was also studied (Figure 3 and Figures S10, S11, Supporting Information). Ni_2_P catalysts chelated by SPhF‐array are consistently better than Ni_2_P, suggesting that SPhF always has a positive effect on both selectivity and activity. And with the increase in SPhF coverage, both TOF and selectivity are improved. Clearly, the best performance is achieved at maximal coverage (33.9%). Compared with reported state‐of‐the‐art catalysts, SPhF‐Ni_2_P gives a much better performance from aspects of conversion and selectivity (Tables S4,S5, Supporting Information). During the recycling test, no obvious activity decay is observed after eight cycles (Figure 3e), and the selectivity is maintained at ≈97%, suggesting a perfect stability of SPhF‐Ni_2_P. And no signal of SPhF is detected in the final product mixture from gas chromatography, further indicating that the strong interaction between SPhF and Ni_2_P ensures the good stability, which can be confirmed by the presence of charateristic peaks of SPhF after the recycling test (Figure S14, Supporting Information).

Above results demonstrate a thiol–Ni_2_P interface significantly boosting the hydrogenation selectivity and activity of Ni_2_P. Then we extend this strategy to commercial Raney Ni catalyst by treating it with SPhF in DMF at 80 °C. As shown in Figure 3f, pristine Raney Ni gives very poor selectivity of 20.5% and 23.4% in hydrogenation of 3‐nitrostyrene and CAL. While for SPhF‐Ni, the selectivity is increased to 100% and 99.2% along with twofold conversions of 41.6% and 48.6%, respectively. Furthermore, this SPhF‐chelated strategy is also extended for commercial Pt/C and Pd/C catalysts. As shown in Figure 3g,h, Pt/C and Pd/C catalysts show unideal selectivity of 74.6% and 45.7%, 55.3% and 46.5% in hydrogenation of 3‐nitrostyrene and CAL. Whereas the selectivity of SPhF‐Pt and SPhF‐Pd is increased to nearly 100% along with nearly twofold conversions. These results further confirm the availability of thiol chelation for promoting the selectivity and activity of hydrogenation catalysts in hydrogenation catalysis.

**Figure 4:**
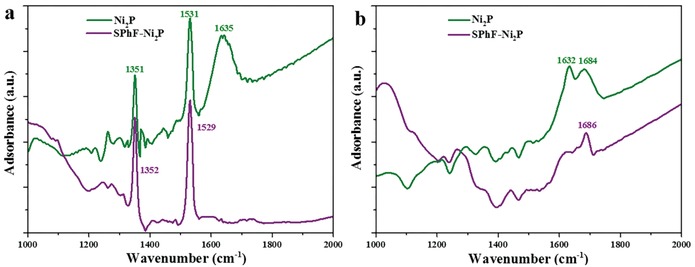
ATR‐IR spectra of a) 3‐nitrostyrene and b) cinnamaldehyde adsorbed on pristine Ni_2_P and SPhF‐Ni_2_P.

Attenuated total reflection infrared spectra (ATR‐IR) spectroscopy results provide further evidence for the switch of hydrogenation selectivity by SPhF chelation, as shown in **Figure** **4**. For Ni_2_P, when 3‐nitrostyrene is used as the probe molecule, several characteristic peaks attributed to the asymmetric and symmetric IR vibrations of –NO_2_ and –C=C at 1531, 1351, and 1635 cm^−1^ can be observed, which reveals 3‐nitrostyrene adsorbs on Ni_2_P via the di‐σ_(ONO)_ and di‐σ_(CC)_ model, suggesting a flat adsorption. In contrast, only vibrations of –NO_2_ appear and no vibrations of –C=C (≈1635 cm^−1^) are present,^2^ indicating that SPhF‐Ni_2_P only absorb nitro group of 3‐nitrostyrene via a vertical configuration. Further styrene adsorption implies SPhF‐Ni_2_P can adsorb the nitro group rather than vinyl group (Figure S15, Supporting Information). In the case of CAL, two inseparable characteristic peaks assigned to –C=C and –CHO appear for Ni_2_P, meaning cinnamaldehyde is adsorbed flatly on Ni_2_P via the di‐σ_(CO)_‐σ_(CC)_. While SPhF‐Ni_2_P presents strong signals assigned to *v*(C=O) and the *v*(C=C) peak becomes faint,^26^, ^43^, ^44^ which means SPhF‐Ni_2_P only absorb –CHO via a vertical configuration (di‐σ_(CO)_), suggesting an inhibited flat adsorption on SPhF‐Ni_2_P.

### 2.3 Modulated d‐Band Center

**Figure 5:**
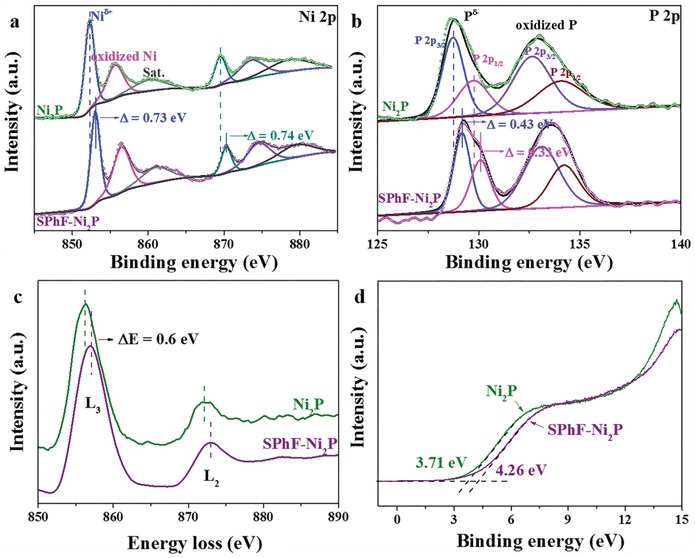
a) Ni 2p XPS spectra and b) P 2p XPS spectra of Ni_2_P and SPhF‐Ni_2_P; c) Ni L‐edge EELS spectra of Ni_2_P and SPhF‐Ni_2_P; d) Valence‐band spectra of Ni_2_P and SPhF‐Ni_2_P measured by UPS.

To understand how surface SPhF affects the electronic structure of Ni_2_P, X‐ray photoelectron spectroscopy (XPS), electron energy loss spectroscopy (EELS), and ultraviolet photoelectron spectroscopy (UPS) were performed. As shown in **Figure** **5**a,b, Ni 2p and P 2p spectra of SPhF‐Ni_2_P exhibit several typical fitted characteristic peaks of Ni_2_P.^26^ S 2p spectra of SPhF‐Ni_2_P shows a symmetrical peak at 163.78 eV assigned to Ni‐S‐R,^36^ suggesting a single chemical environment around S atoms (Figure S16a, Supporting Information). Moreover, the S 2p signal of SPhF‐Ni_2_P disappears after etched by argon sputtering (Figure S16b, Supporting Information), confirming SPhF molecules only exist on Ni_2_P surface. After recycling test, SPhF‐Ni_2_P shows a similar S 2p spectra with the original one, confirming a good stability of SPhF‐Ni_2_P (Figure S17, Supporting Information). Specifically, the binding energies of Ni 2p and P 2p peaks for SPhF‐Ni_2_P both shift positively compared with those of Ni_2_P (≈0.73 and ≈0.74 eV for Ni 2p_3/2_ and Ni 2p_1/2_, ≈0.43 and ≈0.33 eV for P 2p_3/2_ and P 2p_1/2_). In Figure 5c, Ni L‐edge EELS spectra of Ni_2_P and SPhF‐Ni_2_P shows two typical L_2_ and L_3_ peaks. Like the XPS results, a positive shift of ≈0.6 eV is observed for SPhF‐Ni_2_P, suggesting significant deficient charge on Ni_2_P. Figure 5d shows the UPS valence band spectra of Ni_2_P and SPhF‐Ni_2_P, whose valence band maximum values are ≈3.71 eV and 4.26 eV, respectively. Clearly, the valence band is pushed away from the Fermi level after being chelated by SPhF‐arrays. As the d‐band states are determined by the valence charges near the Fermi level, the shift of valence band also implies that the d‐band center of Ni_2_P shifts away from the Fermi level.^26^ Similarly, the SPhE chelation can tune the d‐center of metallic Ni, Pt, and Pd catalysts.^45^, ^46^, ^47^

### 2.4 Role of Surface Chelation by DFT Calculation

**Figure 6:**
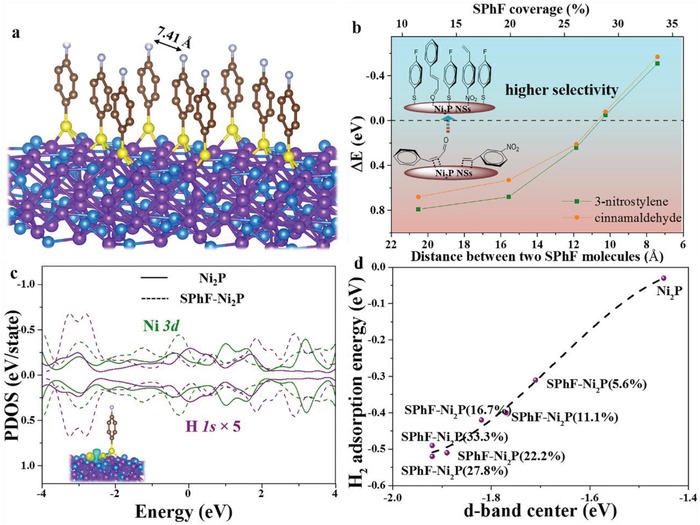
a) Constructed model of SPhF‐Ni_2_P surface (purple, blue, grown, yellow, and white represent Ni, P, C, S, and F, respectively); b) Adsorption exchange energy changes with the distance between two chelated SPhF molecules; c) PDOS plot of Ni_3d_ and H_1s_ orbitals in Ni_2_P and SPhF‐Ni_2_P (insert: differential charge density of H_2_ adsorbed on SPhF‐Ni_2_P interface, yellow and blue indicate electronic charge accumulation and depletion, respectively, with iso‐surface value of 0.008 eÅ^−3^); d) Relationship between H_2_ adsorption energy and d‐band center.

DFT calculation was further applied to uncover the critical role of SPhF on improving the selectivity of Ni_2_P. The stable structures of Ni_2_P (details in Figure S18, Supporting Information) and the surfaces with different coverages of SPhF (details in Figure S19, Supporting Information) were all considered. As shown in Figure S20 in the Supporting Information, on Ni_2_P (001) surface, 3‐nitrostyrene and CAL are adsorbed flatly with large energies of −0.98 and −0.83 eV, respectively, while the vertical adsorption models (with energies of −0.21 and −0.22 eV) are less favorable. That means the hydrogenation of vinyl is thermodynamically preferential over Ni_2_P, in consistent with experimental results. For SPhF‐Ni_2_P, SPhF molecules are adsorbed vertically on Ni_2_P (001) with max coverage, where one S atom binds with three Ni atoms (**Figure** **6**a). Strong electronic coupling along Ni–S bonding is evidenced by the electron charge density redistribution (Figure S21, Supporting Information). The distance between two parallel SPhF molecules (D_SPhF_) is employed to describe the steric effect to evaluate adsorption of reactants. D_SPhF_ values with different SPhF coverages are calculated (Figure S22, Supporting Information); furthermore, the adsorption exchange energy (∆*E* = *E*_ad(vert)_ − *E*_ad(flat)_, *E*_ad(vert)_ and *E*_ad(flat)_ represent vertical and flat adsorption energy) is used as the index to evaluate the priority of vertical adsorption over flat adsorption. Figure 6b shows the change of ∆*E* of 3‐nitrostyrene and CAL with D_SPhF_. ∆*E* changes from positive to negative as D_SPhF_ decreases to less than ≈11 Å, which suggests Ni_2_P with sufficient SPhF coverage (>22.2%) favors vertical adsorption instead of flat one, meaning a switch of hydrogenation selectivity. When SPhF coverage reaches the maximum (33.3%), SPhF molecules can form an ordered 5.93 × 5.93 Å arrays and rhombic‐shaped “channel” (Figure S22, Supporting Information). On account of the confinement effect of SPhF arrays, 3‐nitrostyrene (7.91 Å) and CAL (7.71 Å, Figure S23, Supporting Information) cannot lie flat on Ni_2_P surface but only wedge channels of SPhF‐array with –NO_2_ and –C=O groups interacting with Ni atoms and the C=C group is pushed away from the catalytic sites. Vertical adsorptions via nitro and aldehyde groups are thermally favorable with energies of −0.51 and −0.57 eV (Figure S24, Supporting Information), and increased adsorption energy may be benefited from the van der Waals interactions between parallel benzene rings. Therefore, the flat adsorption of 3‐nitrostyrene and CAL is completely suppressed, confirming the conclusive effect of SPhF confinement in high selectivity. Furthermore, on surfaces of Ni, Pt, and Pd, the ordered SPhF‐arrays also afford a high selectivity to –NO_2_ and –CHO due to the confinement effect.

To investigate the effect of SPhF on the activity of Ni_2_P, Bader charge analysis was performed, showing thiol‐array induces electron‐deficient effect around Ni_2_P (Figure S25, Supporting Information), coinciding with the result of XPS and EELS. Such an electronic transformation is expected to shift d‐band center of Ni away from the Fermi level compared with Ni_2_P (−1.90 vs −1.45 eV) (Figure S26, Supporting Information). Generally, the d‐band center is considered as an important parameter in determining the ability to adsorb H_2_.^18^ Detailed bonding interaction between H_2_ and SPhF‐Ni_2_P interface shows abundant electron accumulation (insertion of Figure 6c), suggesting a strong adsorption of H_2_ over SPhF‐Ni_2_P. Notably, abundant electron transferred from H_2_ to Ni_2_P is observed, suggesting that SPhF‐Ni_2_P is capable of activating H_2_. And projected density of states (PDOS) result exhibits a higher overlap among the binding states between H*_1s_* and Ni_3d_ when H_2_ adsorbs on the surface (Figure 6c), further suggesting a strong adsorption of H_2_. Moreover, with the increase of SPhF coverage, the d‐band center of Ni_2_P continuously downshifts (Figure S26, Supporting Information), and H_2_ adsorption energy depends linearly on d‐band center position (Figure 6d), due to the depressed antibonding energy state.^33^ H_2_ dissociation and atomic H desorption were further computed. Generally, on Ni_2_P (001) surface, H_2_ is heterolysis cleavage with H^δ+^ and H^δ−^ binding with P and Ni,^28^ respectively. Due to negligible adsorption energy of H_2_, Ni_2_P (001) shows a dissociation energy of ≈0.68 eV and unfavorable atomic H desorption (3.62 eV for per H atom), while H_2_ cleavage energy over SPhF‐Ni_2_P becomes negligible (0.08 eV) (Figure S27, Supporting Information). More interestingly, H^δ−^ binding with Ni can easily transfer to nearby S atom with the energy of ≈0.56 eV, accompanied by the breakage of one Ni–S bonding, which significantly accelerates H desorption due to negative‐charged S.

## 3 Conclusion

In summary, we report a facile yet efficient way to boost the performance of nickel‐based catalysts (like Ni_2_P and Raney Ni) for the selective hydrogenation by constructing steric effect and tailoring electronic structure via an organic chelation. The presence of ordered SPhF‐arrays confines the flat adsorption of 3‐nitrostyrene and cinnamaldehyde, by which the hydrogenation of vinyl group is suppressed. Thiol‐array pushes the d‐band center of Ni_2_P away from the Fermi level, promoting the H_2_ molecule activation over Ni_2_P. Compared with Ni_2_P, SPhF‐Ni_2_P exhibits nearly 12 times higher activity and especially its selectivity is increased from 38.1% and 21.3% to nearly 100%. More importantly, such a chelation with organic‐array strategy can be efficiently applied to commercial Raney Ni catalyst, thus providing an easy‐realized and low‐cost way to design catalysts for the selective hydrogenation.

## 4 Experimental Section

*Synthesis of SPhF‐Ni_2_P*: Ni_2_P was phosphatized by Ni(OH)_2_ nanosheets at 325 °C for 1 h under Ar atmosphere using sodium hypophosphite (NaH_2_PO_2_) as phosphorus source (details in Supporting Information). As‐synthesized Ni_2_P (31 mg ) nanosheets was dispersed in 5 mL of DMF in a glass vial. And then: 30 µL of 4‐Fluorothiophenol (SPhF) was added into the mixture and then heated to 80 °C for 12 h. The product was collected by centrifugation with ethanol and dispersed in ethanol for further use. This catalyst is labeled as SPhF‐Ni_2_P and the SPhF coverage is 33.9%. Other catalysts with different SPhF coverages (9.5%, 19.3%, and 26.4%) were also synthesized by altering the dosage of SPhF (7.5, 15, and 22.5 µL), which were named as SPhF‐Ni_2_P (9.5%), SPhF‐Ni_2_P (19.3%), and SPhF‐Ni_2_P (26.4%). Detailed method to determine the SPhF coverage are presented in Table S1 in the Supporting Information.

*Characterizations*: Surface morphologies of catalysts were examined by SEM using a Hitachi S‐4800 instrument. TEM images, selected area electron diffraction, and EELS were obtained using a Tecnai G2 F20 transmission electron microscope at 200 kV, with elemental compositions being analyzed by energy dispersive X‐ray spectrometer (EDXS). The crystal structures were recorded using a RigaKu D/max‐2500 X‐ray diffractometer equipped with a Cu Kα irradiation source. The element analysis was determined by Vario EL CUBE equipped with METTLER x86 instrument. Elemental composition and bonding information were analyzed using an X‐ray photoelectron spectroscopy operated at a pass energy of 187.85 eV (Physical Electronics PHI 1600 ESCA XPS system using a monochromated Al Kα X‐ray source) and the C 1s peak at 284.6 eV was taken as internal standard. Nitrogen adsorption–desorption isotherms were analyzed with ASAP 2020 physisorption Analyzer at 77 K. FT‐IR spectra were recorded on a BioRad FTS 6000 spectrometer. All of the samples were mixed with KBr and pressed into a thin plate for measurement. The S content was determined by inductively coupled plasma optical emission spectroscopy (ICP‐OES) analysis. Before the test, samples were pretreated by microwave.

*Catalytic Hydrogenation*: The hydrogenation was conducted in 50 mL autoclave equipped with a PTFE lining, using 0.5 mmol 3‐nitrostyrene (or cinnamaldehyde), 5 mL solvent, and 10 mg Ni_2_P. The autoclave was purged with N_2_ and H_2_ (15 bar) respectively for three times, pressurized to 10 bar, and heated to 70 °C for defined time. After the reaction, the autoclave was cooled to room temperature, followed by centrifugation and analysis of a sample by GC‐MS (Agilent 5975 equipped with HP‐5 capillary column) and GC (Agilent 7820 equipped with FID detector and AT‐SE‐54 capillary column). When Raney Ni and SPhF‐Ni were used as catalysts, the reaction condition was quite similar to the case of Ni_2_P except for the reaction temperature of 120 °C. The kinetic analysis was performed at the range of low conversion (0–80 min reaction for 3‐nitrostyrene and 1.5 h reaction for cinnamaldehyde). TOF values were measured at 1 h and calculated by Equation (1) and based on each exposed Ni atom uncovered by SPhF

(1) $$\mathrm{TOF}\;=\;\frac{n_{0}C}{tn_{\mathrm{cat}}}$$

where *n*_0_ is the initial molar of substrate, *C* is the conversion of substrates at the reaction of *t*, and *n*_cat_ is the molar of exposed Ni atom determined by Table S1 in the Supporting Information.

*Density Functional Theory Calculations*: All density functional theory calculations were carried out with Vienna Ab‐initio Simulation Package (VASP).^48^, ^49^ The exchange correlation functional employed was the Perdew–Burke–Ernzerhof (PBE) functional of generalized gradient approximation (GGA).^50^, ^51^ To locate transition states (TS), the climbing images nudged elastic band (CI‐NEB) algorithm was used.^52^ Each transition state was confirmed by vibrational frequency analysis. The calculations employed DFT‐D to estimate the van der Waals interactions among adsorbates.^13^, ^53^ The vacuum region was 20 Å. The valence electrons were described by a plane wave basis set with the kinetic energy cutoff of 500 eV, and the core electrons were replaced by the projector augmented wave (PAW) pseudopotentials. The k‐point was set to 2 × 2 × 1 and that of static method was 6 × 6 × 1. A four‐layer slab with a 4 × 8 supercell was built, and the top two layers were relaxed. All the structures were optimized until the force on each atom was less than 0.02 eV Å^−1^. The adsorption energies, *E*_ads_, were calculated by using Equation (2)

(2) $$E_{\mathrm{ads}}\;=\;E_{\mathrm{adsorbate+surface}}\;-\;\left(E_{\mathrm{adsorbate}}\;+\;E_{\mathrm{surface}}\right)$$

where *E*_ads_ is the adsorption energy, *E*_adsorbate+surface_ is total energy of surface covered with adsorbates, *E*_surface_ is the energy of clean surface, and *E*_adsorbate_ is the energy of adsorbate. The d‐band center, ξ_d_ was calculated as the first and second moments of the projected d‐band density of states^54^ by using Equation (3)

(3) $$\xi d\;=\frac{\int_{-\infty }^{+\infty }\rho EdE}{\int_{-\infty }^{+\infty }\rho dE}$$

where ρ represents the density of states and *E* represents the energy of state.

## Conflict of Interest

The authors declare no conflict of interest.

## Supporting information

Supplementary
