# Supplementary material for: Breaking Trade‐Off between Selectivity and Activity of Nickel‐Based Hydrogenation Catalysts by Tuning Both Steric Effect and d‐Band Center
Source: Adv Sci (Weinh). 2019 Mar 25;6(10):1900054. doi: 10.1002/advs.201900054 (PMC6523378; doi:10.1002/advs.201900054)
Supplement: Supplementary file 1 — Supplementary [file ADVS-6-1900054-s001.pdf]

Copyright WILEY-VCH Verlag GmbH & Co. KGaA, 69469 Weinheim, Germany, 2019.

ADVANCED SCIENCE
Open Access

Supporting Information

for *Adv. Sci.,* DOI: 10.1002/advs.201900054

Breaking Trade-Off between Selectivity and Activity of Nickel-Based Hydrogenation Catalysts by Tuning Both Steric Effect and d-Band Center

*Ruijie Gao, Lun Pan, Huiwen Wang, Yunduo Yao, Xiangwen Zhang, Li Wang, and Ji-Jun Zou**

WILEY-VCH

Copyright WILEY-VCH Verlag GmbH & Co. KGaA, 69469 Weinheim, Germany, 2019.

Supporting Information

Breaking Trade-Off between Selectivity and Activity of Nickel-Based Hydrogenation Catalysts by Tuning both Steric Effect and d-Band Center

Ruijie Gao, Lun Pan, Huiwen Wang, Yunduo Yao, Xiangwen Zhang, Li Wang, Ji-Jun Zou*

Supplemental Experimental procedures

Chemicals.

All chemicals (AR grade) were used as received without any further purification. Water with a resistivity of 18.2 MΩ·cm was used for all experiments. All glassware was thoroughly washed by 1M hydrochloric acid to avoid any possible contamination.

Synthesis of hierarchical Ni_2P nanosheets. Synthesis of hierarchical Ni_2P nanosheets involves two steps: synthesis of hierarchical $Ni(OH)_2$ and following phosphatization. The preparation of $Ni(OH)_2$ nanosheets was performed as follows. Typically, 0.24 mmol of $NiCl_2$ and 4.8 mmol of urea were dissolved in 100 mL of distilled water. After the mixture was stirred for 10 min, the mixed solution was kept at 90 ^oC for 48 h. Finally, the precipitates were washed three times with water and ethanal, respectively, and dried at 80 ^oC for 6 h to obtain $Ni(OH)_2$ nanosheets. To synthesize Ni_2P nanosheets, 200 mg of sodium hypophosphite (NaH_2PO_2) was put in the uptake and 30 mg of $Ni(OH)_2$ was put in the downtake, and then calcined at 325 ^oC for 1 h under Ar atmosphere to realize phospharization of nickel. After that, the powders were washed for 3 times using deionized water to remove unconverted sodium hypophosphite, and then dried under 60 ^oC.

Supplemental Characterization.

Dynamic CO pulsed chemisorption was also performed on a Micromeritics ASAP 2920 automatic analyzer equipped with a TCD. The passivated catalyst sample (50 mg, maintained at room temperature for 3 h in 0.5 wt.% O_2/N_2) was loaded into a quartz reactor and reduced in H_2 flow (99.999%, 100 mL/min STP) at 500 ^oC for 2 h with a ramp of 10 ^oC/min. Then the sample was purged with He (99.999%, 50 mL/min STP) at 500 ^oC for 2 h and cooled to 35 ^oC under a He flow. The Co pulse was repeatedly injected until the response from the detector showed no further CO uptake after consecutive injections.

The attenuated total reflection infrared (ATR-IR) spectroscopy was acquired with a Bruker Equinox 55 spectrometer equipped with a DLaTGS detector and operated at room temperature and atmospheric pressure. Before the test, the catalyst was dispersed into the ethanol and treated with ultrasonic for 30 min. then, the suspension was dropwise added onto the surface of the diamond crystal equipped on the instrument at the reaction temperature. After about 2 hours, the catalyst dried at 90 ^oC. Here, the background spectrum was recorded before the substrate solution was added.

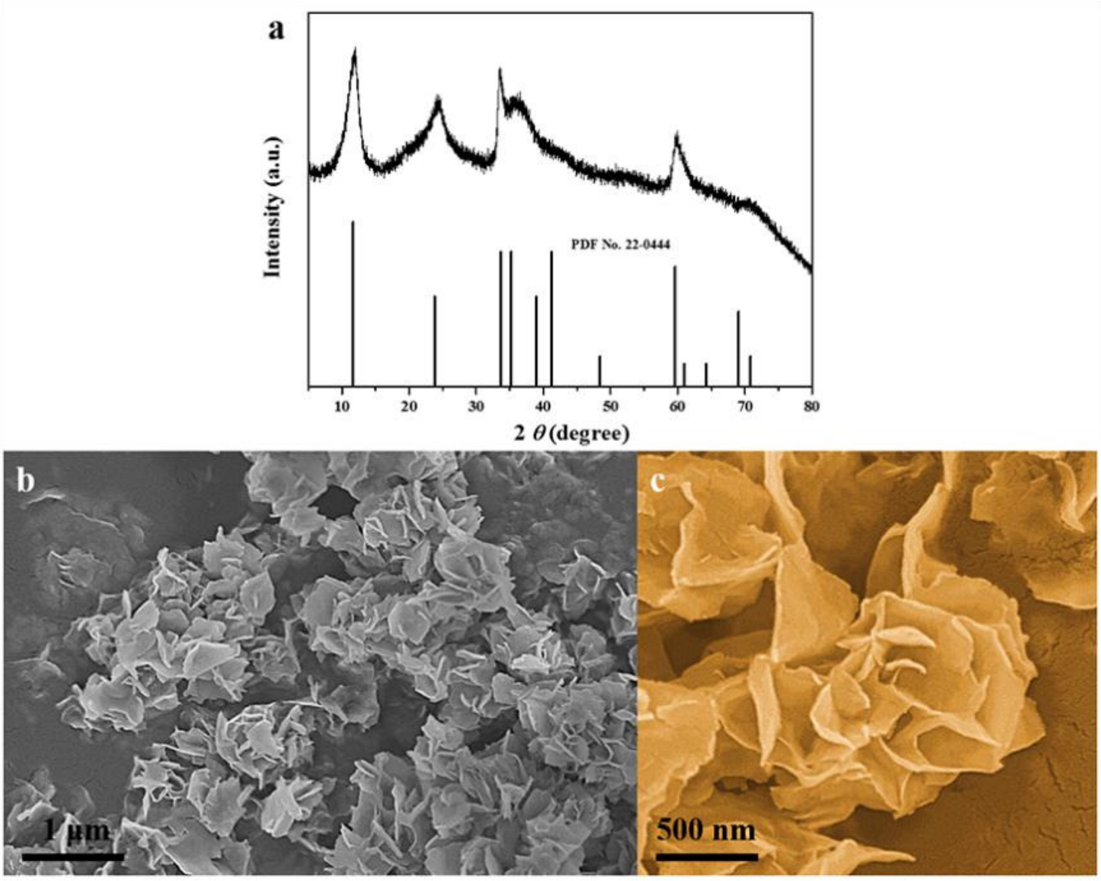

Figure S1. (a) XRD pattern and (b)(c) SEM images of $Ni(OH)_2$ NSs.

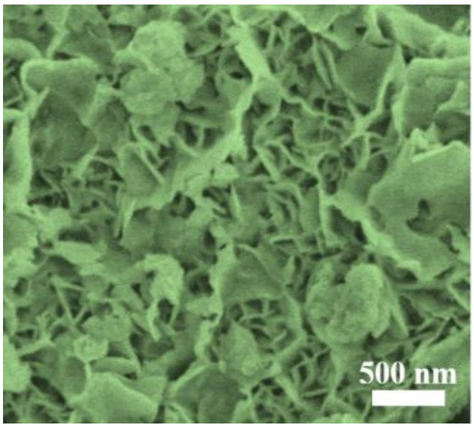

Figure S2. SEM image of Ni_2P.

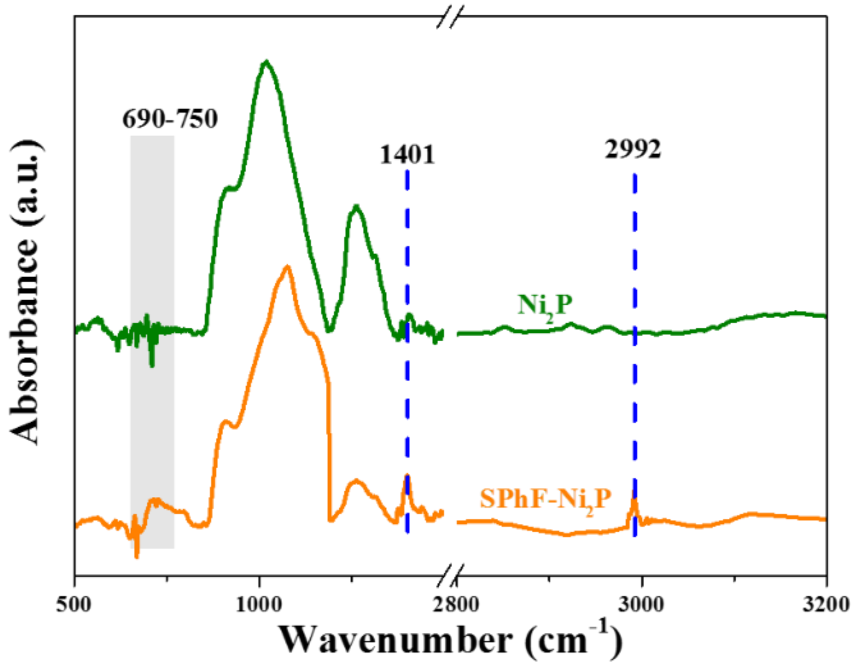

Figure S3. FT-IR spectra of pristine Ni_2P and SPhF-Ni_2P.

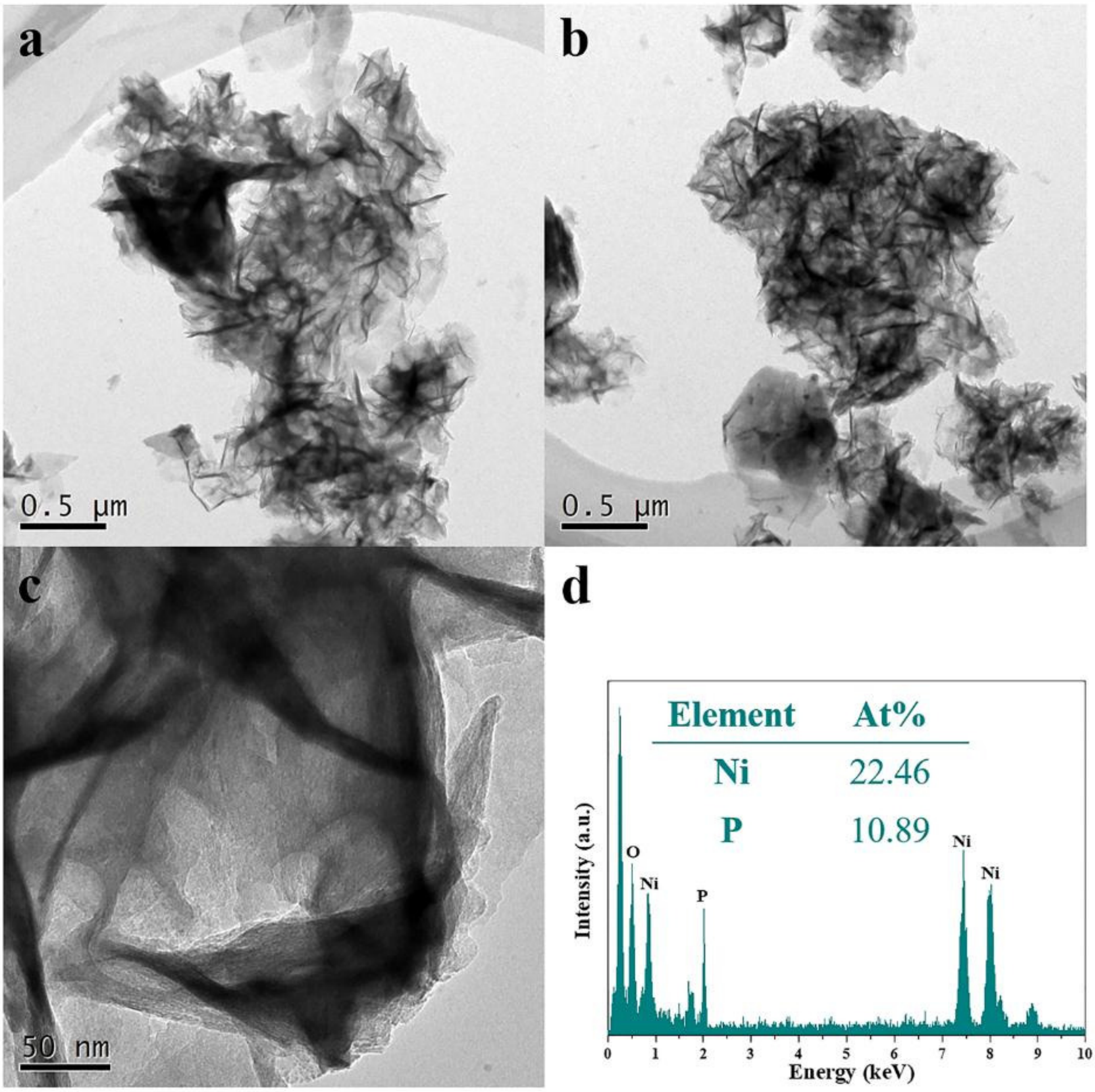

Figure S4. (a), (b), and (c) TEM images of Ni_2P NSs. (d) TEM-EDX spectrum of Ni_2P NSs.

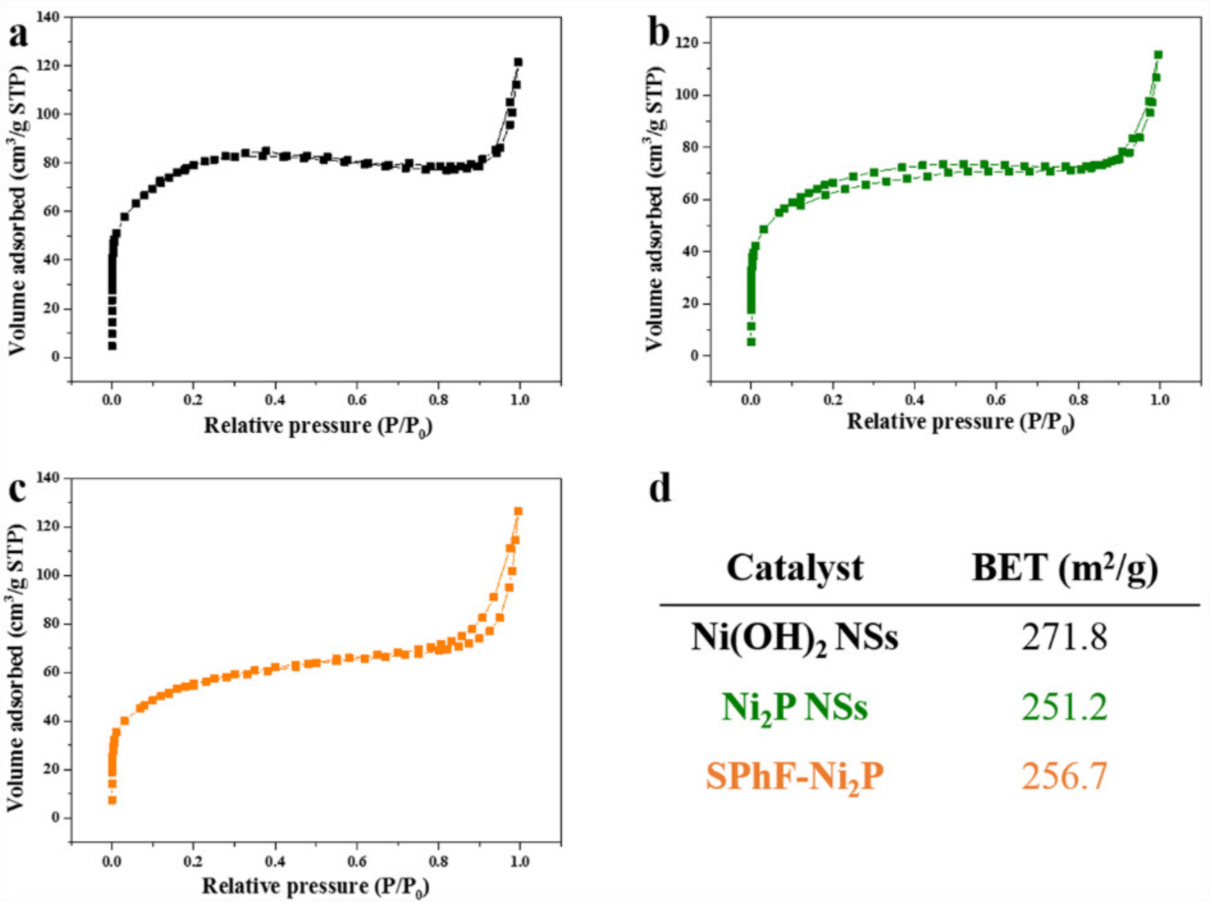

Figure S5. N_2 adsorption/desorption isotherm plots of (a) $Ni(OH)_2$ NSs, (b) Ni_2P NSs, and (c) SPhF-Ni_2P. (d) the corresponding BET areas.

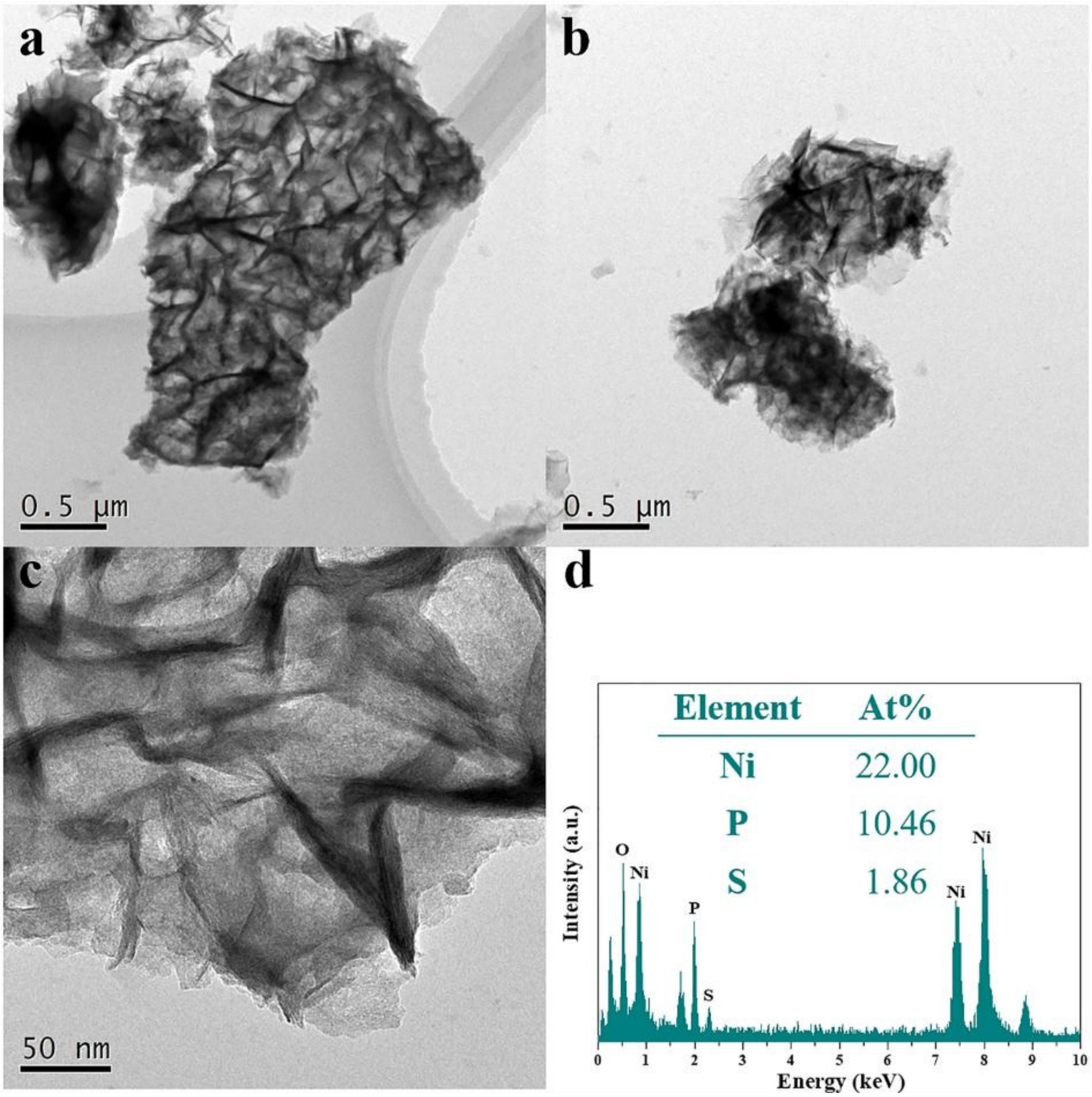

Figure S6. (a), (b), and (c) TEM images of SPhF-Ni_2P. (d) TEM-EDX spectrum of SPhF-Ni_2P.

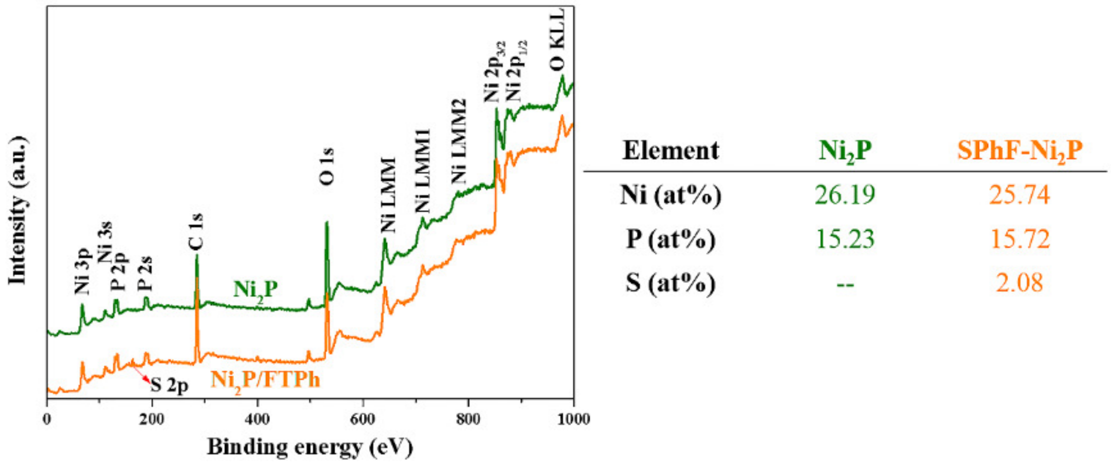

Element	Ni_2P	SPhF-Ni_2P
Ni (at%)	26.19	25.74
P (at%)	15.23	15.72
S (at%)	--	2.08

Figure S7. XPS survey spectra of Ni_2P and SPhF-Ni_2P and the corresponding element concentration.

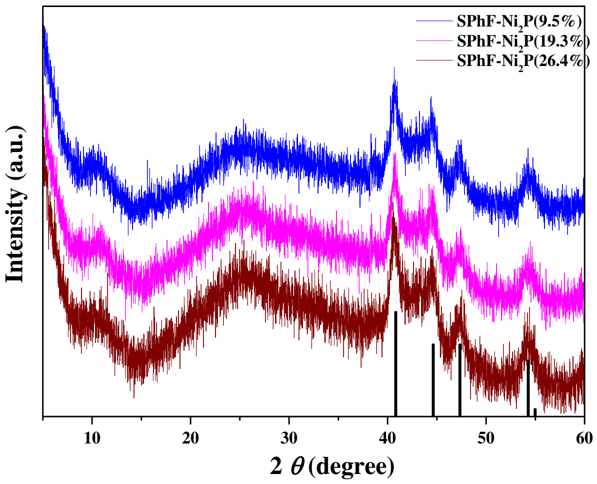

Figure S8. XRD patterns of SPhF-Ni_2P (9.5%), SPhF-Ni_2P (19.3), and SPhF-Ni_2P (26.4%), respectively.

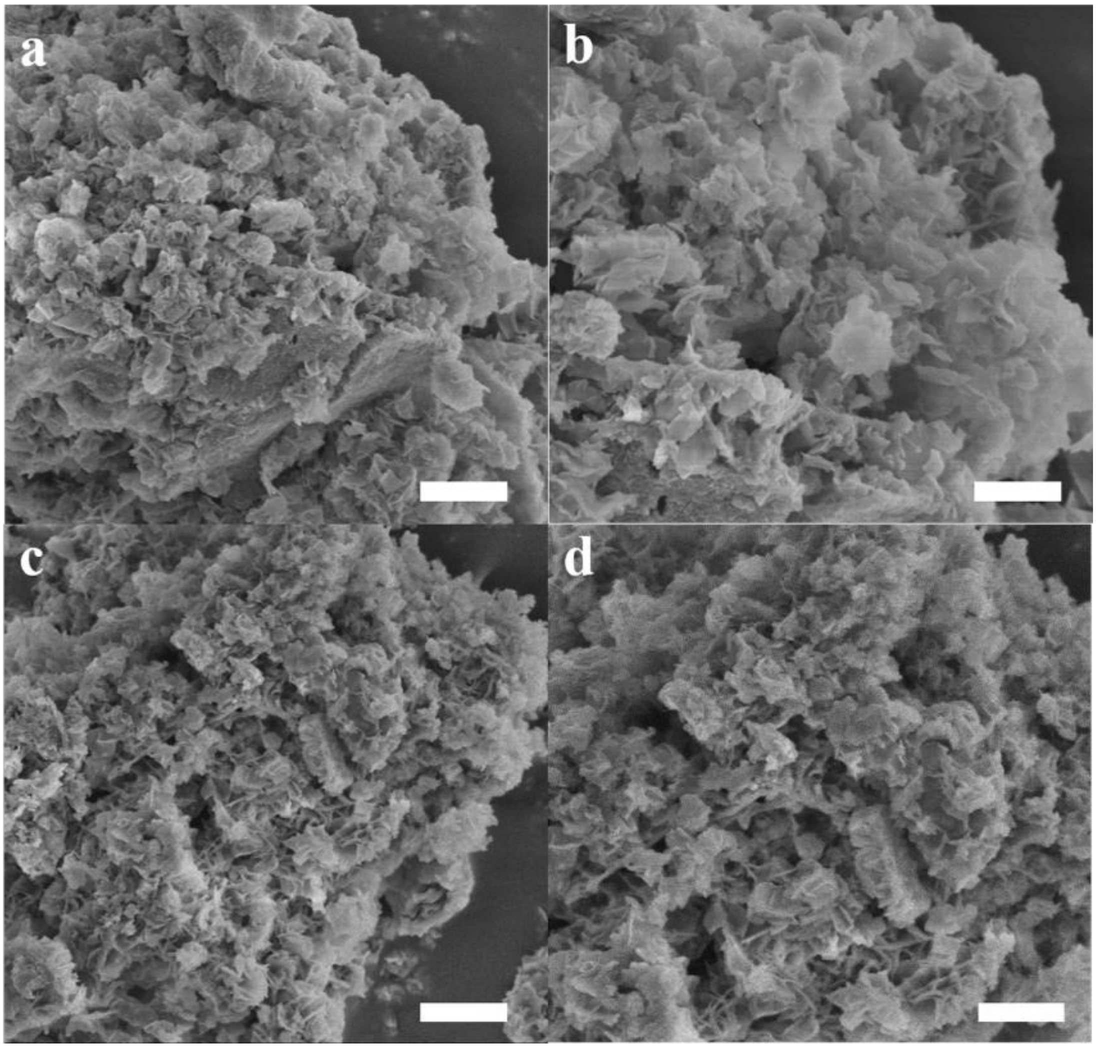

Figure S9. SEM images (scale bar 2 μm) of (a) SPhF-Ni_2P(9.5%), (b) SPhF-Ni_2P(19.3), (c) SPhF-Ni_2P(26.4%), and (d) SPhF-Ni_2P.

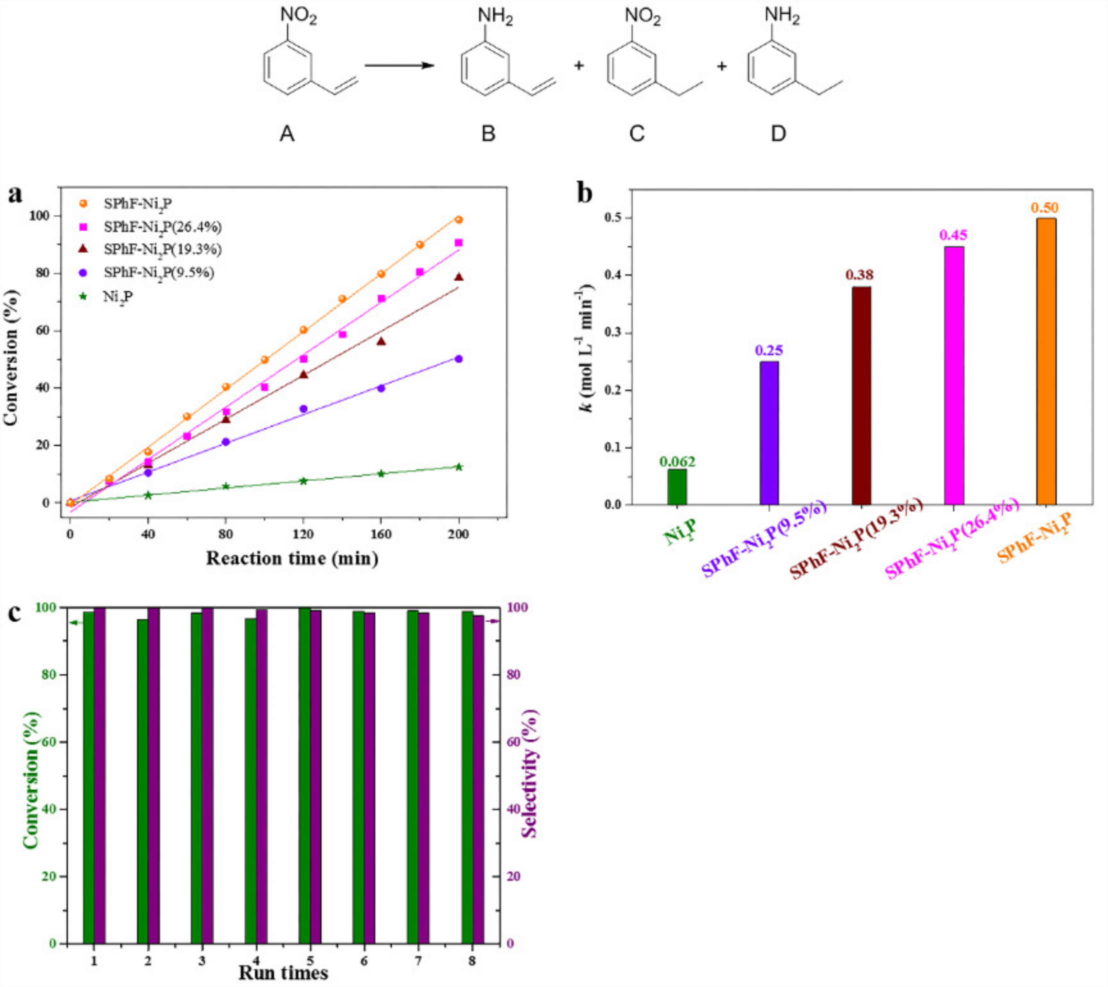

Figure S10. Selective hydrogenation of 3-nitrostyrene over Ni_2P and SPhF-Ni_2P catalysts. (a) Conversion of 3-nitrostylene against reaction time; (b) Fitted reaction rate constants based on conversion of 3-nitrostylene; (c) Recycling test of SPhF-Ni_2P for hydrogenation of 3-nitrostylene.

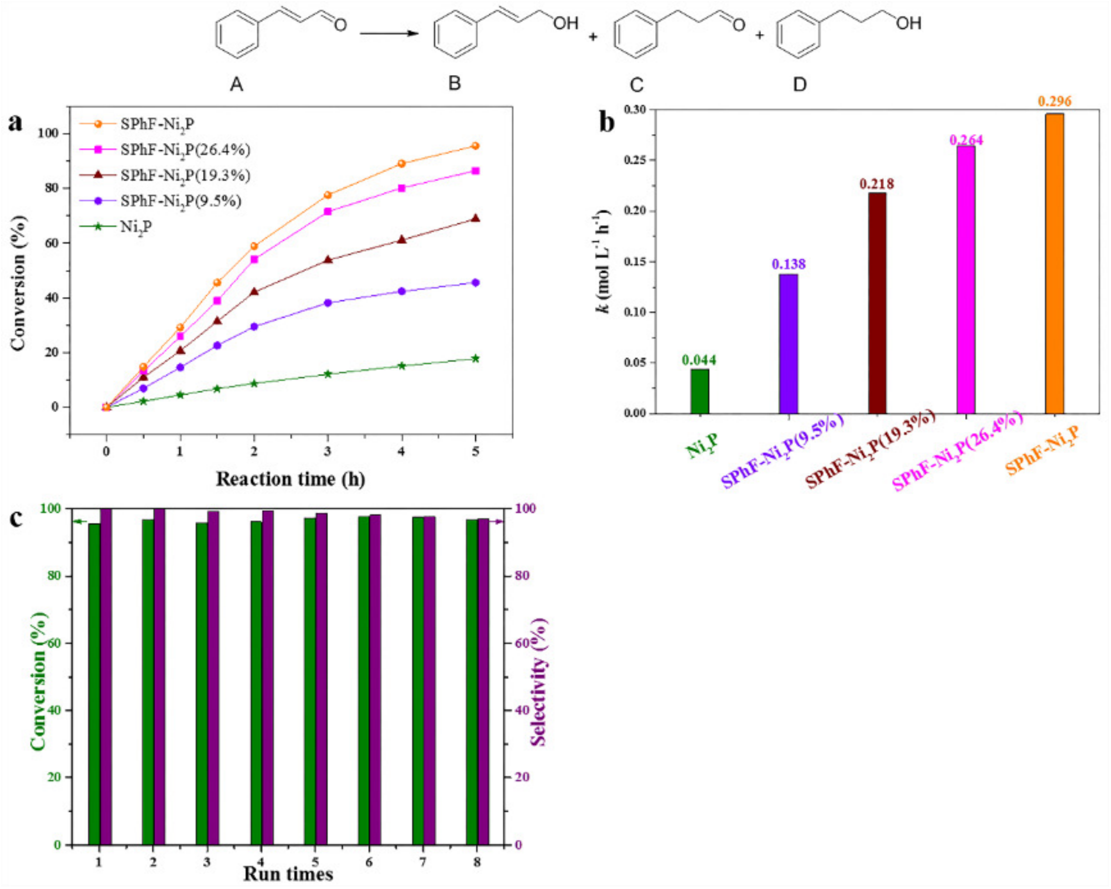

Figure S11. Selective hydrogenation of cinnamaldehyde over Ni_2P and SPhF-Ni_2P catalysts. (a) Conversion of cinnamaldehyde against reaction time; (b) Fitted reaction rate constants based on conversion of cinnamaldehyde; (c) Recycling test of SPhF-Ni_2P for hydrogenation of cinnamaldehyde.

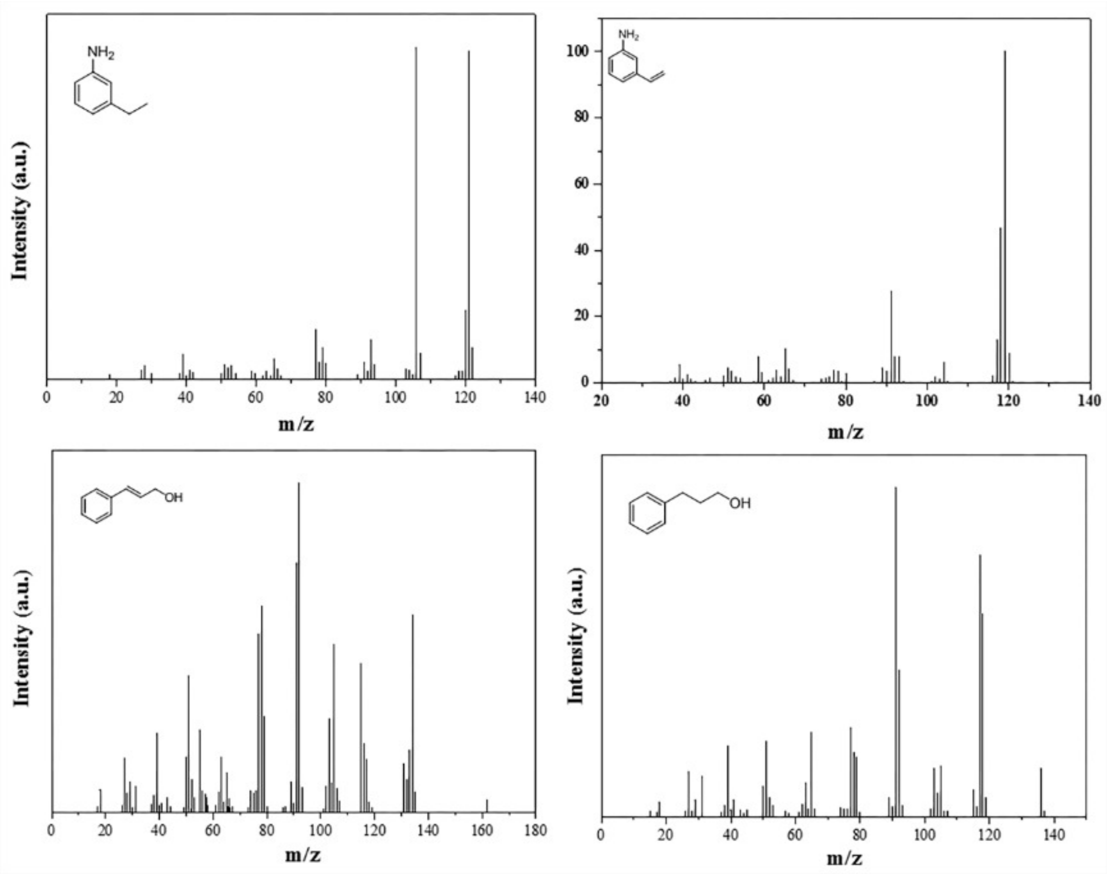

Figure S12. Supplemental MS spectra of products.

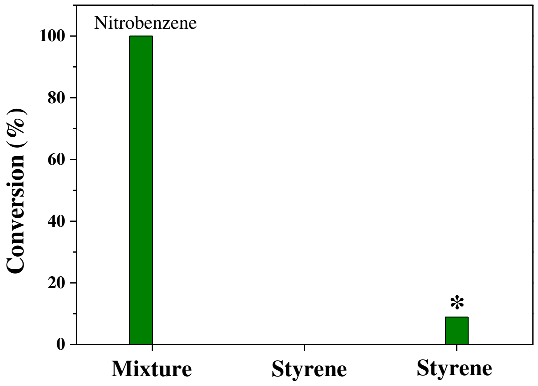

Figure S13. Competitive reaction over SPhF-Ni_2P. Solo styrene and mixture of nitrobenzene and styrene are used as substrates over SPhF-Ni_2P. Reaction conditions: 70 ^oC, 10 bar hydrogen, 10 mg catalyst, 5 mL ethanol. Substrates: 0.5 mmol styrene or mixture (0.25 mmol nitrobenzene and 0.25 mmol styrene). *temperature: 120 ^oC.

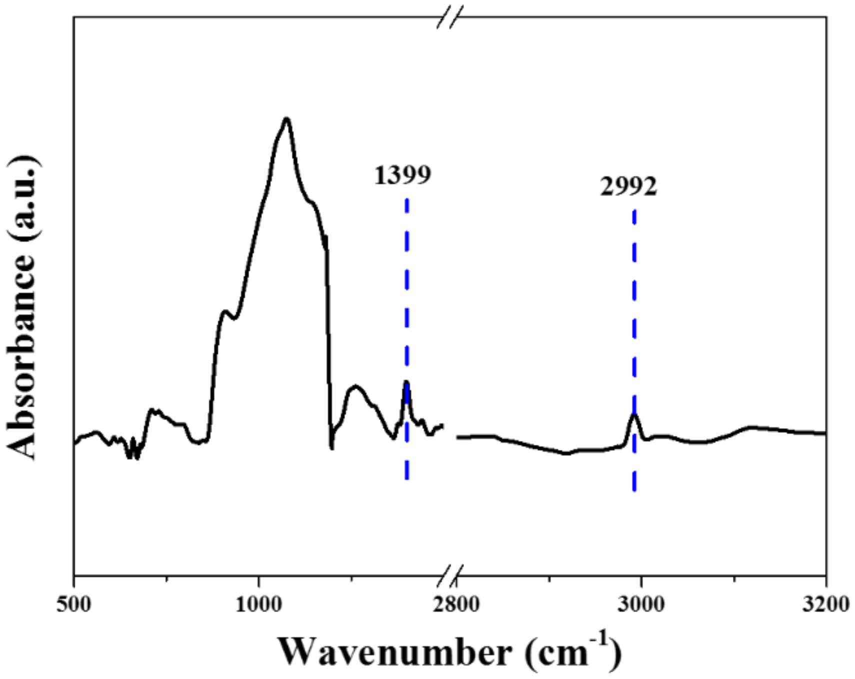

Figure S14. FT-IR spectra of SPhF-Ni_2P after the recycling test.

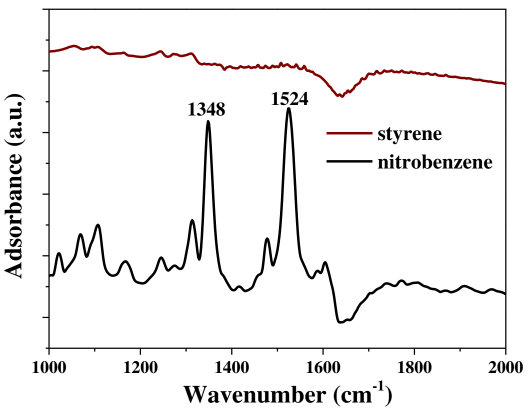

Figure S15. FT-IR spectra of solo styrene and nitrobenzene over SPhF-Ni_2P.

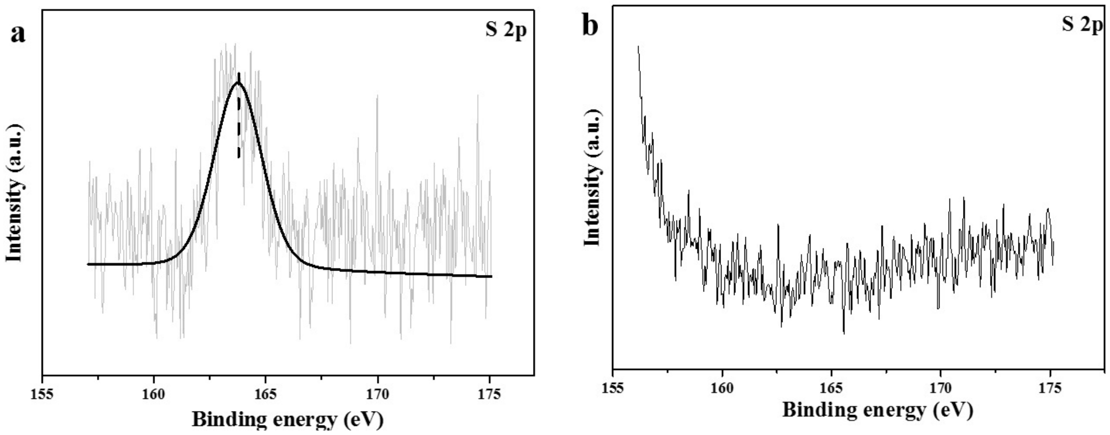

Figure S16. S 2p XPS spectra of (a) SPhF-Ni_2P and (b) SPhF-Ni_2P etched by argon-ion for 30 seconds.

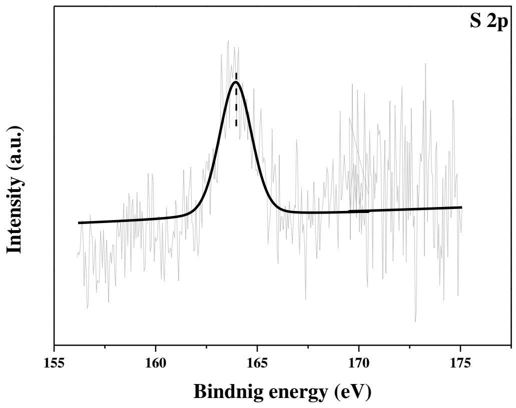

Figure S17. S 2p XPS spectra of SPhF-Ni_2P after recycling test.

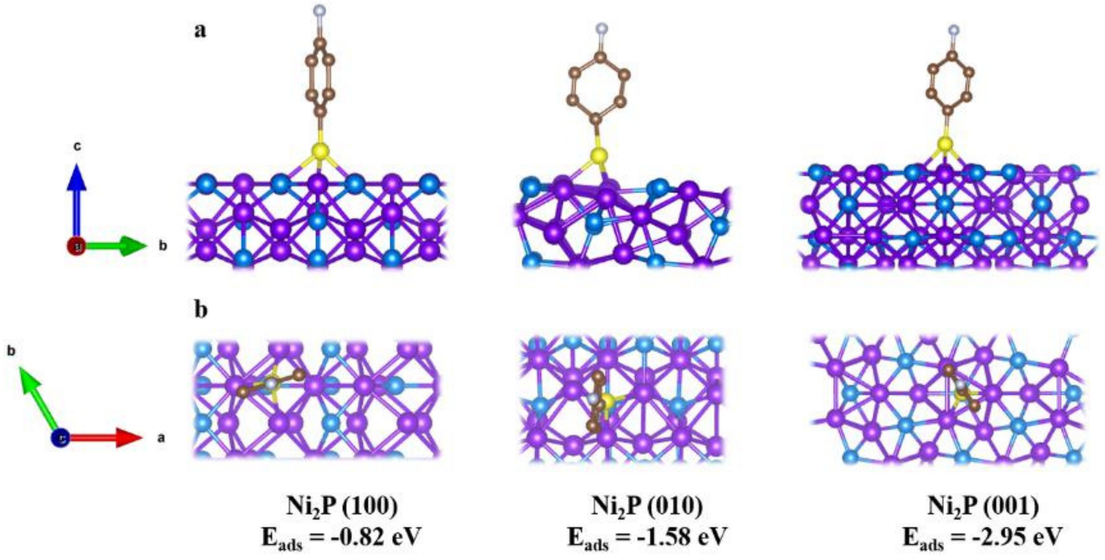

Figure S18. SPhF adsorption models on different Ni_2P surfaces and the corresponding adsorption energies.

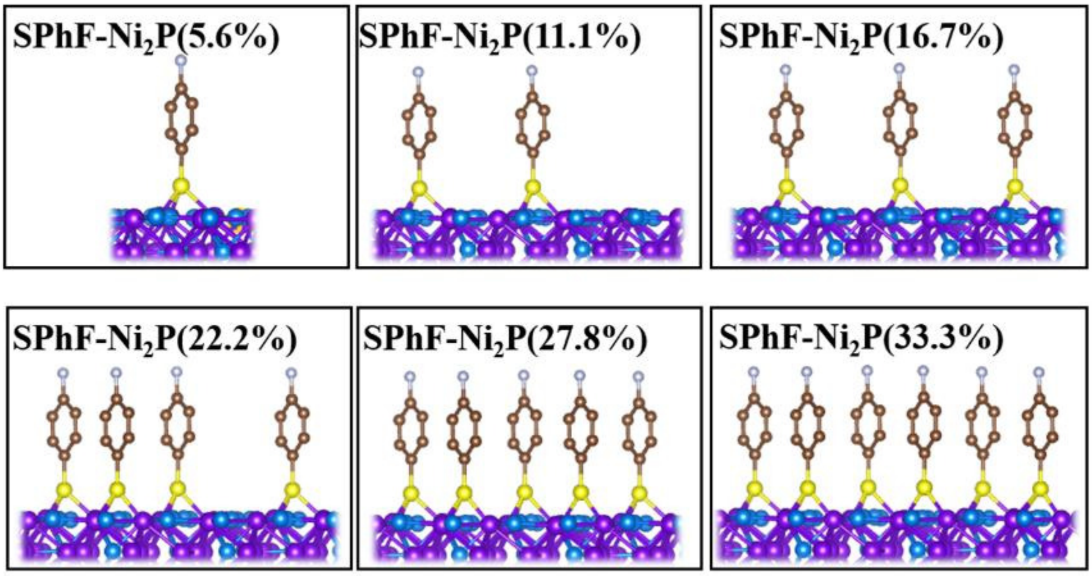

Figure S19. Adsorption models of different SPhF coverages on Ni_2P (001).

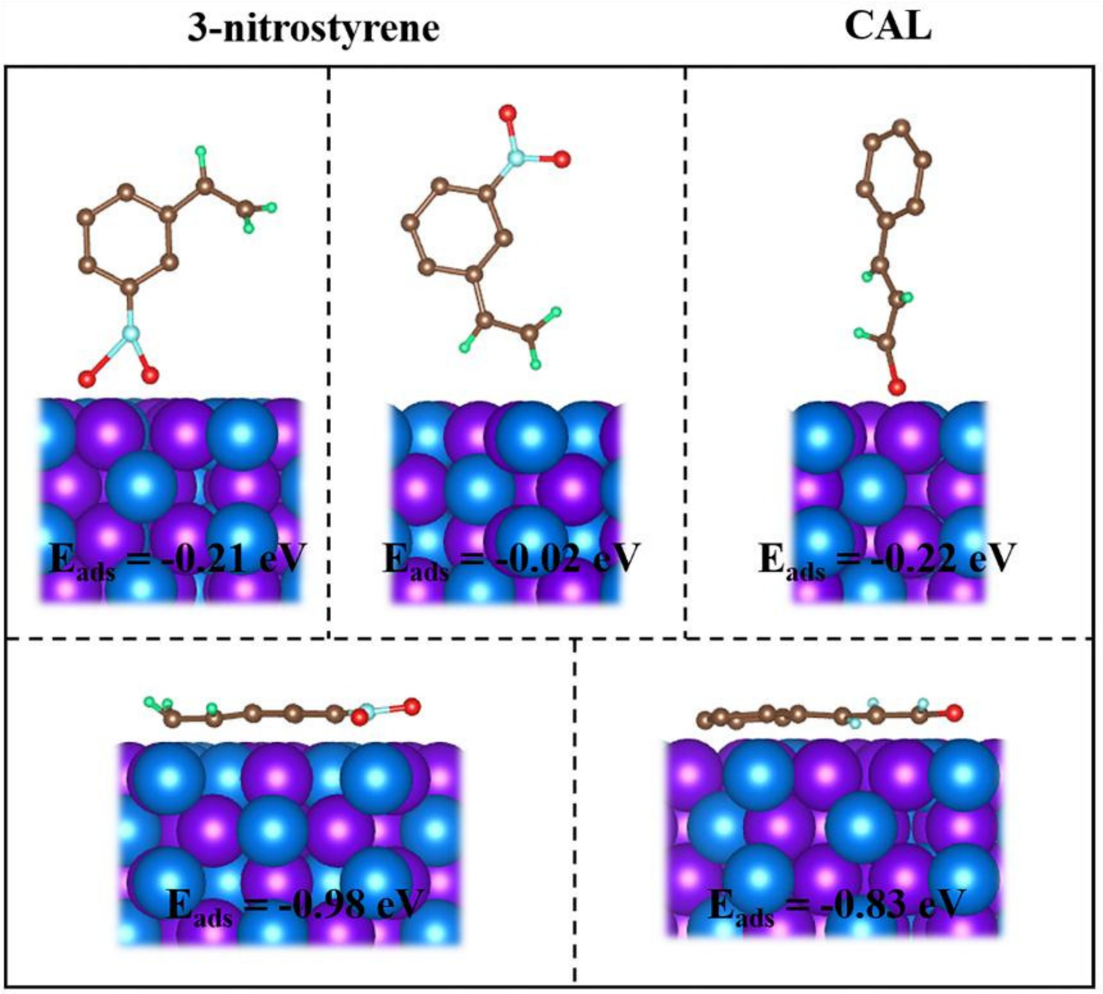

Figure S20. Different adsorption models of 3-nitrostylene and cinnamaldehyde on Ni_2P (001) surface and the corresponding adsorption energies.

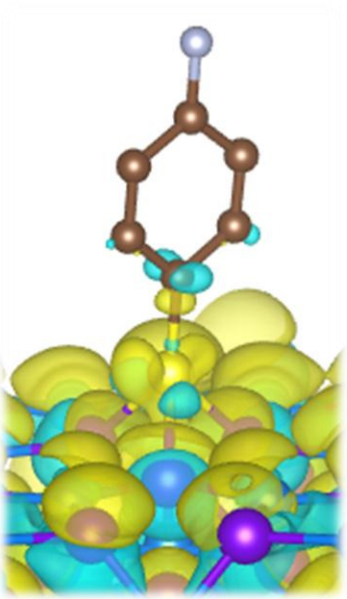

Figure S21. Differential charge density of SPhF adsorbed on Ni_2P (001) surface (yellow and blue indicate electronic charge accumulation and depletion, respectively, with iso-surface value of 0.002 eÅ^{-3}).

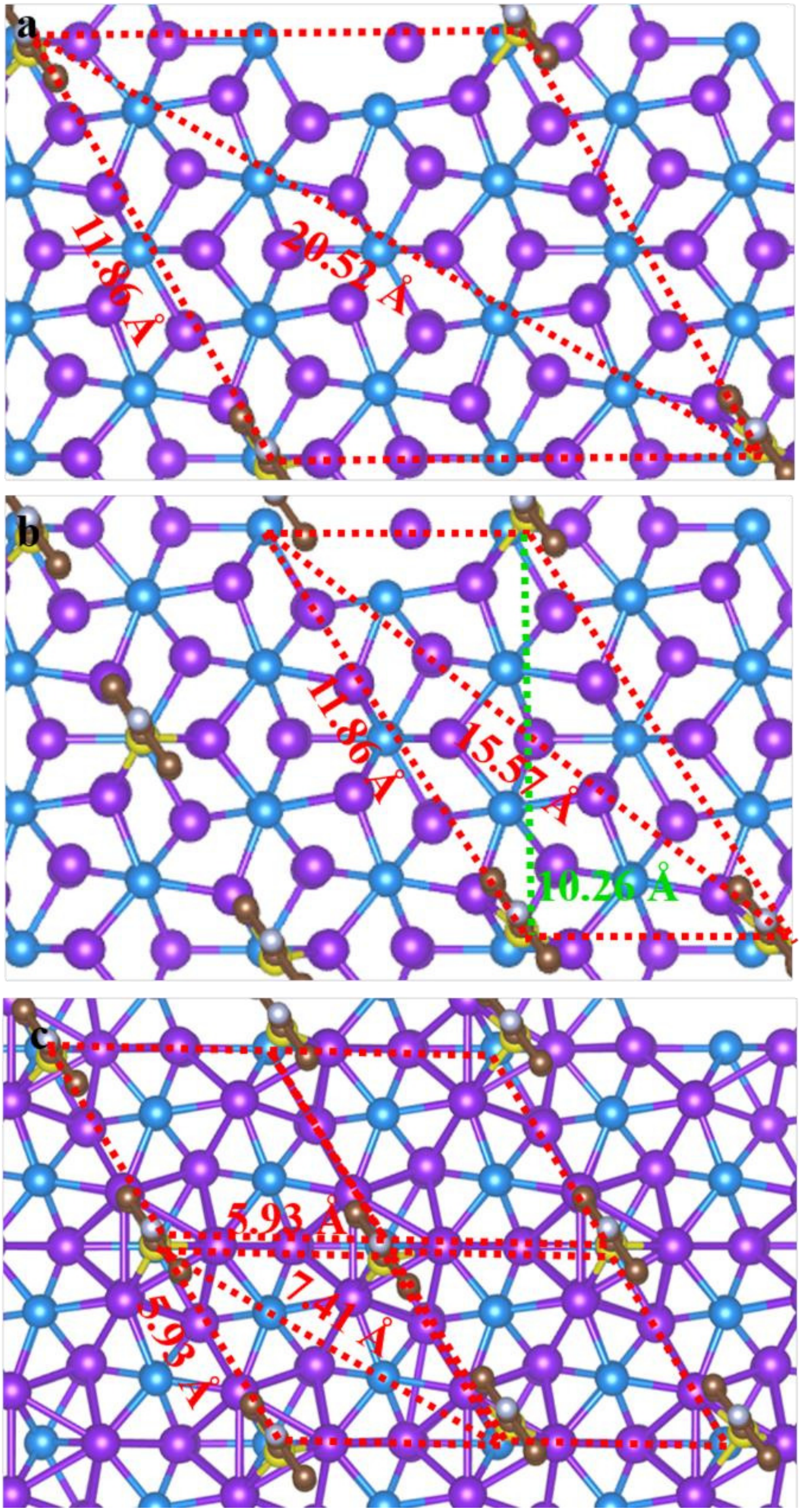

Figure S22. Proposed different distances between two adsorbed SPhF molecules on (a) Ni_2P/SPhF(11.1%), (b) Ni_2P/SPhF(22.2%) and (c) Ni_2P/SPhF(33.3%)

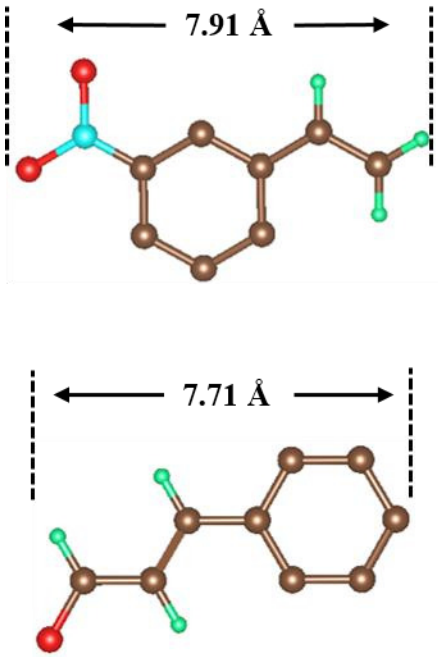

Figure S23. Relaxed 3-nitrostyrene and CAL molecules (red, cyan and green represent O, N and H atoms, respectively) and the corresponding length (key bond length: $R_{C\text{-}O}$ = 1.51 Å; $R_{C\text{-}C}$ = 1.54 Å; $R_{C\text{-}N}$ = 1.51 Å; $R_{N\text{-}O}$ = 1.48 Å).

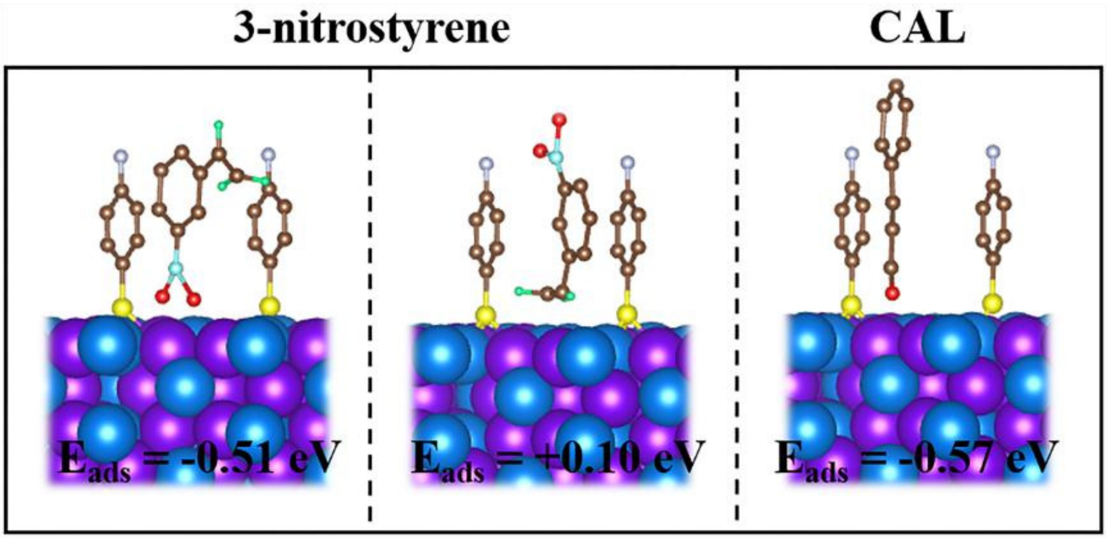

Figure S24. Different adsorption models of 3-nitrostylene and cinnamaldehyde on SPhF-Ni_2P interface and the corresponding adsorption energies.

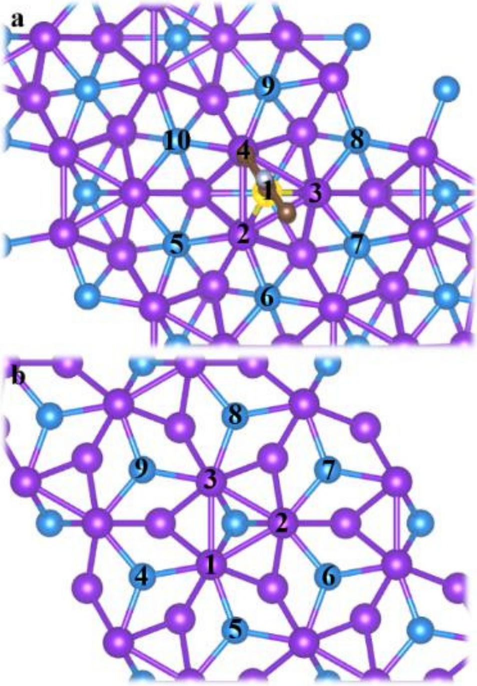

atom	charge (e)	atom	charge (e)
1	6.24	6	5.21
2	9.79	7	5.21
3	9.79	8	5.21
4	9.79	9	5.21
5	5.21	10	5.21

atom	charge (e)	atom	charge (e)
1	9.88	6	5.25
2	9.87	7	5.25
3	9.87	8	5.25
4	5.25	9	5.25
5	5.25		

Figure S25. Bader charge analysis for (a) SPhF-Ni_2P and (b) Ni_2P (purple and blue represent Ni and P atoms, respectively).

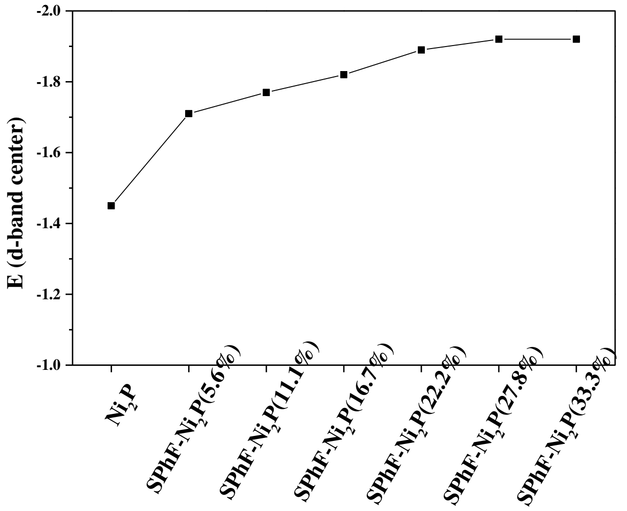

Figure S26. The d-band center of the functionalized Ni atoms as the function of SPhF coverage.

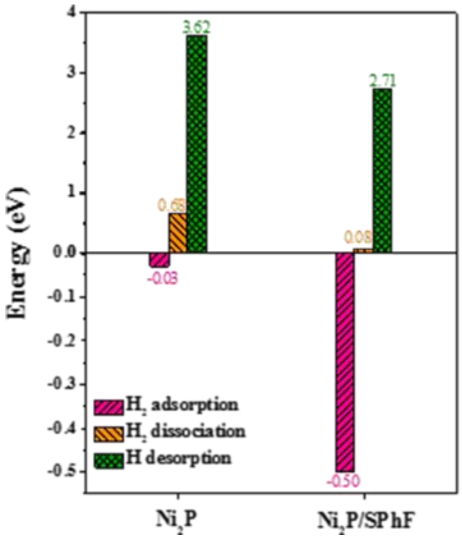

Figure S27. Energies of H_2 adsorption, dissociation, and desorption for Ni_2P and SPhF-Ni_2P(33.3%).

Table S1. The SPhF coverage of SPhF-Ni_2P catalysts.

	CO uptake (μmol/g)	Coverage (%)
Ni_2P	254	/
	S concentration (mg/g)	
SPhF-Ni_2P(9.5%)	0.77	9.5
SPhF-Ni_2P(19.3%)	1.57	19.3
SPhF-Ni_2P(26.4%)	2.15	26.4
SPhF-Ni_2P	2.76	33.9

Note: we hypothesis that CO molecules are adsorbed on Ni_2P with the mono layer, therefore CO uptake can be regarded as the exposed number of Ni. The SPhF coverage is calculated as following equation : $C_{SPhF} = \frac{C_S}{C_{CO}} \times 100\%$ (C_{SPhF}, C_S, C_{CO} represent the SPhF coverage, S concentration, and CO uptake, respectively).

Table S2. The optimization of reaction condition for selective hydrogenation of 3-nitrostyrene.

Entry	Solvent	Time (min)	Temperature (°C)	Pressure (MPa)	Conversion (%)	Selectivity (%)
1	Ethanol	40	70	1.0	17.8	>99.9
2	Cyclohexane	40	70	1.0	3.7	>99.9
3	H_2O	40	70	1.0	28.6	91.2
4	THF	40	70	1.0	6.8	>99.9
5	Ethanol	40	80	1.0	22.6	>99.9
6	Ethanol	40	90	1.0	27.4	>99.9
7	Ethanol	40	100	1.0	36.8	>99.9
8	Ethanol	40	70	1.5	19.5	>99.9
9	Ethanol	40	70	2.0	22.2	>99.9
10	Ethanol	40	70	2.5	25.1	>99.9
11	Ethanol	40	70	3.0	30.6	>99.9
12	Ethanol	900	70	1.0	100	98.8
13	Ethanol	600	120	1.0	100	97.1

Reaction condition: 10 mg catalyst, 0.5 mmol 4-nitrobenzaldehyde, 5 mL solvent.

Table S3. The optimization of reaction condition for selective hydrogenation of cinnamaldehyde.

Entry	Solvent	Time (min)	Temperature (°C)	Pressure (MPa)	Conversion (%)	Selectivity (%)
1	Ethanol	60	80	1.0	29.2	>99.9
2	Cyclohexane	60	80	1.0	5.5	>99.9
3	H_2O	60	80	1.0	40.6	91.2
4	THF	60	80	1.0	9.8	>99.9
5	Ethanol	60	90	1.0	37.6	>99.9
6	Ethanol	60	100	1.0	44.5	>99.9
7	Ethanol	60	110	1.0	53.1	>99.9
8	Ethanol	60	80	1.5	29.5	>99.9
9	Ethanol	60	80	2.0	32.1	>99.9
10	Ethanol	60	80	2.5	34.2	>99.9
11	Ethanol	60	80	3.0	39.9	>99.9
12	Ethanol	900	80	1.0	100	99.0
13	Ethanol	600	120	1.0	100	95.8

Reaction condition: 10 mg catalyst, 0.5 mmol 4-nitrobenzaldehyde, 5 mL solvent.

Table S4. Selective hydrogenation of 3-nitrostyrene using different catalysts.

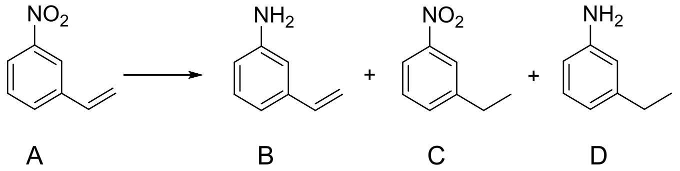

Catalyst	T (^oC)	Con. (%)	Yield (%) B	Yield (%) C	Yield (%) D	Ref.
SPhF-Ni_2P	70	100	99.0	0	1.0	**This work**
Au_{25}/ZnAl-HT-300	90	100	99.0	1.0	0	*Angew. Chem. Int. Ed.* **2017**, *56*, 2709-2713.
1.5 wt%Au-TiO_2	90	100	86.3	12.6	1.1	*Angew. Chem. Int. Ed.* **2017**, *56*, 2709-2713.
1 wt% Pt/TiO_2	80	100	0	32.0	62.0	*ACS Catal.* **2012**, *2*, 2079-2081.
Pd/C	120	99.0	0	23.8	75.1	*Science* **2006**, *313*, 332-334.
Pt/C	120	96.7	2.8	50.6	43.3	*Science* **2006**, *313*, 332-334.
Ni_2P/PC-2	80	96.1	99.0	1.0	0	*ACS Catal.* **2018**, *8*, 8420-8429.
CoS_2/PC	110	>99.0	97.0	0	3.0	*Green Chem.* **2018**, *20*, 671.
$LaCu_{0.67}Si_{1.33}$	120	95.0	100	-	-	*J. Am. Chem. Soc.* **2017**, *139,* 17089-17097.

Table S5. Selective hydrogenation of cinnamaldehyde using different catalysts.

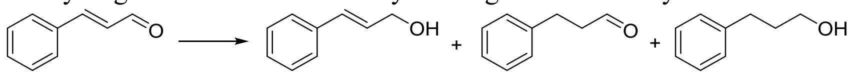

A B C D

Catalysts	T (^{o}C)	Con. (%)	Yield (%) B	Yield (%) C	Yield (%) D	Ref.
SPhF-Ni_2P	80	100	98.8	0	1.2	**This work**
$PtNi_{2.20}$ NWs@Ni/Fe_4-MOF	40	99.5	83.3	15.1	1.65	*Small* **2018**, *14*, 1704318.
MIL-101(Fe)@Pt@MIL-101(Fe)$^{9.2}$	25	94.3	97.0	3.0	0	*Nature* **2016**, *539*, 76.
MIL-101(Cr)@Pt@MIL-101(Fe)$^{2.9}$	25	99.8	95.6	3.6	0.8	
Pt/Graphene	60	92.0	88.0	7.0	3.0	*ChemCatChem* **2014**, *6*, 3246.
Pt@Uio-66-NH_2	25	85.9	87.9	-	-	*ACS Catal*. **2014**, *4*, 1340.
Pt/MgAl-LDH	60	79.7	85.4	1.0	13.3	*Catal. Sci. Technol.* **2013**, *3,* 2819.
PdSn/AC	130	96.0	78.0	-	-	*Catal. Commun.* **2014**, *43,* 102.
NiCo/MWCNT	150	30.0	69.0	0	31.0	*Ind. Eng. Chem. Res.* **2014**, *53,* 13910.
CuAu/SiO_2	100	65.0	65.0	13.0	22.0	*AIChE J.* **2014**, *60,* 3300.
CuCr/SBA-15	150	11.0	52.0	18.0	30.0	*Catal. Sci. Technol.* **2013**, *3,* 2319.
Au/$MgAlCeO_2$	120	91.0	44.0	14.0	41.0	*Ind. Eng. Chem. Res.* **2013**, *52,* 288.
Pt_3Ni@$Ni_{32}Cu(OH)_2$-2 NWs	50	98.5	4.5	87.9	7.6	*Adv. Funct. Mater.* **2018**, *28,* 1705918.
